# *Escherichia coli* can survive stress by noisy growth modulation

**DOI:** 10.1038/s41467-018-07702-z

**Published:** 2018-12-17

**Authors:** Om Patange, Christian Schwall, Matt Jones, Casandra Villava, Douglas A. Griffith, Andrew Phillips, James C. W. Locke

**Affiliations:** 1 Sainsbury Laboratory, University of Cambridge, Cambridge, CB2 1LR UK; 2 Department of Biochemistry, University of Cambridge, Cambridge, CB2 1QW UK; 3 Microsoft Research, Cambridge, CB1 2FB UK

## Abstract

Gene expression can be noisy, as can the growth of single cells. Such cell-to-cell variation has been implicated in survival strategies for bacterial populations. However, it remains unclear how single cells couple gene expression with growth to implement these strategies. Here, we show how noisy expression of a key stress-response regulator, RpoS, allows *E*. *coli* to modulate its growth dynamics to survive future adverse environments. We reveal a dynamic positive feedback loop between RpoS and growth rate that produces multi-generation RpoS pulses. We do so experimentally using single-cell, time-lapse microscopy and microfluidics and theoretically with a stochastic model. Next, we demonstrate that *E*. *coli* prepares for sudden stress by entering prolonged periods of slow growth mediated by RpoS. This dynamic phenotype is captured by the RpoS-growth feedback model. Our synthesis of noisy gene expression, growth, and survival paves the way for further exploration of functional phenotypic variability.

## Abstract (web summary)

Noisy gene expression leading to phenotypic variability can help organisms to survive in changing environments. Here, Patange et al. show that noisy expression of a stress response regulator, RpoS, allows *E*. *coli* cells to modulate their growth rates to survive future adverse environments.

## Introduction

The phenotype of organisms can vary due to changes in the genome arising from mutations. The role of such genotypic variation and its influence on evolution has been well studied^1^. Less is known about phenotypic variability arising from stochastic processes affecting gene regulatory dynamics and the function of such variability. Examples of noisy gene expression used to prepare for changing environments have been found in diverse organisms. Several bacterial species have been found to use noise to evade antibiotics^2–5^ and overcome nutrient limitation^6–8^ without the need to mutate. Higher organisms can also use phenotypic variability to handle environmental fluctuations; examples include yeast^9–11^, multicellular fungi^12^, and plants^13^.

The pervasiveness of noisy gene expression lies in its origin. It arises from the random collisions of small concentrations of regulators, polymerases, and nucleic acids in cells^14–17^. Indeed, many genes tested in *E*. *coli* exhibit variability^18,19^. Gene regulatory networks could evolve to either suppress such noise to improve robustness of critical phenotypes^20^, or to amplify it to generate a range of transcriptional states in individual cells. Recent work has found the latter case to exist and has revealed pulsatile gene expression dynamics as a mechanism to enhance variability^6,21,22^. Furthermore, noise is not isolated to expression of single genes, but has been found in bacterial physiology as well. This is remarkable since a physiological process such as growth is the product of many genes. Yet, noisy growth rates have been widely observed in bacteria^6,7,23–25^.

**Fig. 1:**
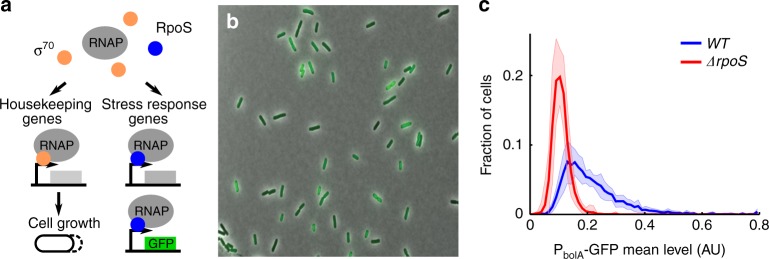
The stress response master regulator, RpoS, is heterogeneously expressed in unstressed cells. **a** Schematic of the role of sigma factors σ^70^ and RpoS in promoting growth and activation of the stress response regulon, respectively. Also illustrated is the RpoS reporter, a transcriptional fusion to a stress response promoter. **b** Representative phase contrast and fluorescence composite image of RpoS reporter, *P*_*bolA*_*-GFP*, in *WT*; channel ranges chosen for display. **c** Histograms of mean GFP per cell (line: mean, shaded region: ± std dev) in *WT* (10 biological replicates, 4037 cells, mean = 0.21, CV = 0.51) and *ΔrpoS* (9 bio. reps., 4069 cells, mean = 0.11, CV = 0.27) strains. The long tail of high RpoS expression present in the *WT* is absent in the knockout

We used the stress response system of *E*. *coli* as a model to study how noisy gene expression and noisy growth rates might couple to produce functional phenotypic variability. *E*. *coli* respond to stress by expressing a range of protective genes. Global stress response is controlled, in large part, by RpoS (also known as σ^S^ and σ^38^), which is an alternative sigma factor^26,27^. Sigma factors are a component of the RNA polymerase holoenzyme that recognise and bind to the promoter region of genes^27^. The housekeeping sigma factor, σ^70^, promotes the transcription of genes responsible for growth, for instance ribosomal genes^28^. Conversely, RpoS upregulates stress response genes^26,28^ (Fig. 1a). RpoS is strongly upregulated in the transition from exponential to stationary phase when cells are starved for resources^29^. Populations in exponential phase have also been shown to express small amounts of functional RpoS^30,31^. However, these studies were of bulk cultures, which can mask single cell phenotypes.

Here we take a single-cell approach to uncover heterogeneous expression of RpoS in exponential phase *E*. *coli*^32^. We reveal the pulsatile dynamics that give rise to RpoS heterogeneity by tracking single cells over many generations in the Mother Machine microfluidic device^33^. Using modelling^14,34^ and experiments, we show that RpoS and growth rate are coupled in a mutual inhibition feedback loop and that this coupling gives rise to the RpoS pulses. Finally, we demonstrate that a function of this coupling of noisy gene expression and growth rate is to allow *E*. *coli* to survive sudden stress.

## Results

### RpoS is heterogeneously distributed at the single-cell level

The low RpoS expression observed by others in exponential phase cells^30,31^ prompted our first question: How is this RpoS distributed amongst single cells? It could be that all cells have basal levels of RpoS or some cells could express the majority of the RpoS. To answer this question we grew cells in bulk culture into exponential phase and examined aliquots of the culture with single cell resolution under a microscope^32^ (see Fig. 1b and Methods). As a proxy for RpoS we used a transcriptional reporter with a promoter from an RpoS-responsive gene fused to GFP: *P*_*bolA*_*-GFP* (Fig. 1a)^28,35^. By computing histograms of mean GFP level per cell we discovered that RpoS is heterogeneously distributed amongst single cells (Fig. 1c). We found RpoS to be similarly distributed in a strain where the only source of RpoS was from a translational fusion of RpoS to mCherry: *rpoS::mCherry* (Supplementary Figure 1a, b). However, the translational fusion was not able to activate the transcriptional reporter, suggesting that the fluorescent protein fusion disrupts RpoS function (Supplementary Figure 1c). To test the transcriptional fusion further we carried out the same liquid culture assay on an *rpoS*-knockout (*ΔrpoS*, Fig. 1c)^36^. The characteristic long tail of the heterogeneous *WT* distribution vanished in the knockout strain, leaving only spurious gene expression^28,37^. Our imaging assay may have caused the heterogeneity in RpoS expression by inducing a stress response, so we fixed cells while still in liquid culture (see Methods section). This did not eliminate the long-tail of the *WT* distribution, despite potential denaturation of the GFP by the fixation process^38^, suggesting the heterogeneity is intrinsic to the liquid culture (Supplementary Figure 2a). Moreover, we found similar behaviour when alternative reporters for RpoS were tested (Supplementary Figure 2b, c)^28^. To test whether the long-tail was specific to RpoS, we examined σ^70^ reporters. The distributions of σ^70^ levels in *WT* populations had less pronounced long-tails due to the higher abundance of σ^70^ in cells and did not change significantly in *ΔrpoS* (Supplementary Figure 3)^28^.

### RpoS pulsing produces heterogeneous population

**Fig. 2:**
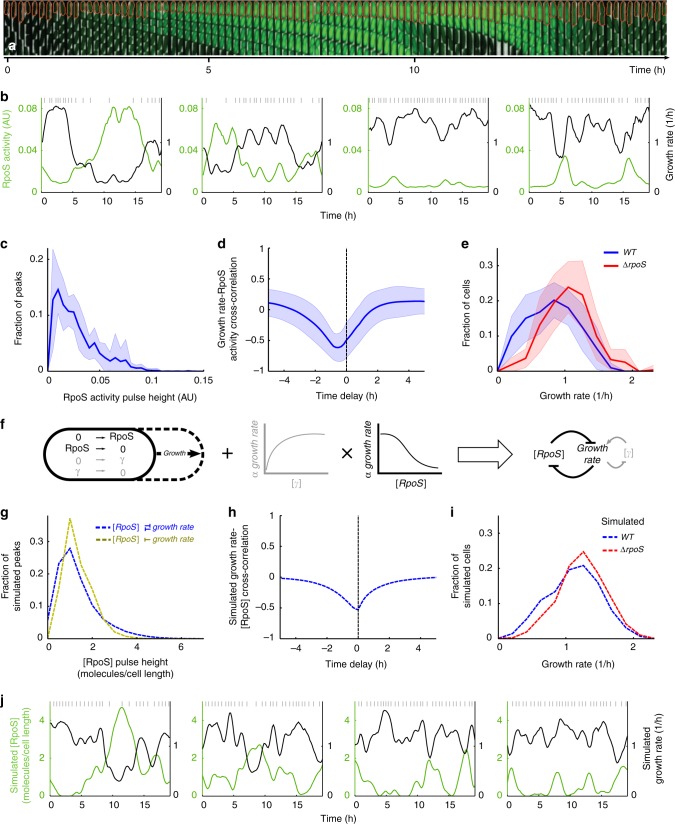
Growth-RpoS mutual inhibition produces multi-generation RpoS pulses and heterogeneous RpoS expression. **a** Sample montage of a mother cell (orange outline) in the Mother Machine pulsing on RpoS and reducing growth rate (1 frame/10 min). Phase contrast and fluorescence channel ranges chosen for display. **b** Sample time traces of RpoS activity and growth rate for four mother cells. Grey vertical lines indicate cell divisions. **c** Histogram of RpoS activity pulse height (3378 peaks). **d** Cross-correlation between growth rate and RpoS activity. **e** Histogram of growth rate at one frame (59) from all movies for *WT* (505 cells) and *ΔrpoS* (272 cells). In **c**–**e** the mean ± std dev is plotted with the line and the shaded region, respectively, for *WT* (11 technical replicates drawn from 7 biological replicates, 507 mother cells) and *ΔrpoS* (10 tech. reps. drawn from 6 bio. rep., 274 mother cells). **f** Schematic illustration of mathematical model. Stochastic molecular reactions occur in a growing cell. The reactions are simulated with the Gillespie algorithm, while cell growth happens at deterministic time steps. Growth at each time step is dependent on molecular concentration via Hill functions. The result is a mutual inhibition between growth rate and RpoS concentration. **g**–**j** Analysis from 1000 simulations run for 500 h; only the last 250 h are used to avoid initial transients in the simulation. **g** Histograms of simulated RpoS concentration with and without feedback of RpoS on growth rate (88,865 and 133,126 pulses, respectively). **h** Cross-correlation between simulated growth rate and RpoS concentration. **i** Histograms of growth rate sampled at 24 h intervals over all 1000 simulations. **j** Sample time traces of simulated RpoS concentration and growth rate for four cells. Grey vertical lines indicate cell divisions

We next investigated the mechanism by which the RpoS distribution is produced. Reasoning that the distribution is due to a dynamic equilibrium, not a fixed subpopulation, we tracked single cells over multiple generations using time-lapse microscopy^32^ and the Mother Machine microfluidic device^33^ (Fig. 2a, Methods, Supplementary Movie 1). We found cells had heterogeneous P_bolA_-GFP levels in this environment as well (Supplementary Figure 1h). By computing the rate of production of the GFP signal, we extracted the RpoS activity (see Methods section for derivation and Supplementary Figure 4). Indeed, we found rich, dynamic RpoS activity. A small fraction of cell lineages have high RpoS activity pulses lasting multiple generations while others have a range of pulse sizes, including very small pulses (Fig. 2b, Methods section). For example, 8% of cell lineages have at least one RpoS activity peak above 0.08 AU (Fig. 2b). To test whether the pulsing was an artefact of the Mother Machine environment we grew cells in an alternative microfluidic device as well as on agarose pads^32^ containing media and found similar pulsing behaviour (see Methods section, Supplementary Figure 5, Supplementary Movies 2, 3). We also observed similar dynamics with alternative RpoS transcriptional reporters in the Mother Machine (Supplementary Movies 4, 5).

Analysing the *P*_*bolA*_*-GFP* Mother Machine data, we found a long-tailed distribution of pulse heights. This supported the idea that the long-tailed liquid culture distribution is generated by cells pulsing RpoS on to different levels (Fig. 2c). The *rpoS::mCherry* translational fusion also had a long tailed distribution of pulse heights in the Mother Machine, validating these RpoS dynamics (Supplementary Figure 1d–g). However, again, the translational fusion was not able to transcriptionally activate *P*_*bolA*_*-GFP* under our conditions (Supplementary Figure 1h, i). We chromosomally integrated the *P*_*bolA*_*-GFP* reporter and found a similar consistency between bulk culture and microfluidic experiments suggesting the dynamics did not arise due to plasmid segregation noise (Supplementary Figure 6a–c). However, the fluorescence signal was very dim, thus we proceeded with the plasmid-based reporter.

### Single cell growth rate is also noisy

We further observed rich dynamics in the growth rate of single cells in the Mother Machine experiments (Fig. 2b; Supplementary Figures 4a, b, and 6a; and Methods section). The sample lineages illustrate that cell growth slows down when RpoS activity is high. This relationship was quantified as a large negative value near zero time-shift in the cross-correlation of growth rate and RpoS activity (Fig. 2d, Supplementary Figure 6e, Methods). The strong anti-correlation suggested that growth rate should also be widely distributed, which is what we observed (Fig. 2e, Supplementary Figure 6d, 7b). However, the *ΔrpoS* strain also had a wide growth rate distribution suggesting growth rate is intrinsically heterogeneous^23^ (Fig. 2e, Supplementary Figure 7b). Furthermore, σ^70^ activity was positively correlated with growth rate suggesting it is related to this intrinsic variability (Supplementary Figure 7a).

### Coupled molecular and physiological model captures observations

We propose a coupled molecular and physiological model to explain our observations. First, we propose the intrinsic variability in growth rate arises due to stochastic molecular reactions that promote growth. Second, we propose that RpoS molecules repress growth and that growth dilutes RpoS. This results in the anti-correlation between growth rate and RpoS.

To test our proposal we constructed a mathematical model. For simplicity, we chose to model two molecular species, growth factor *(γ)* and RpoS *(r)*. We used a stochastic Gillespie simulation for the reactions^14,34^. Both were assumed to be produced by zeroth order reactions and degraded by first order reactions (Fig. 2f, see Methods section for details). The reactions occurred in a cell, which grew at deterministic time intervals. As the cell volume increased, molecule concentration of both RpoS and *γ* was diluted. The growth rate at each deterministic time step explicitly depended on the most recent *γ* and RpoS concentration via the product of Hill functions (Fig. 2f). The Hill function for *γ* rose with concentration while that for RpoS decreased. This captured the promoting and repressing effects on growth rate of the two kinds of molecules, respectively.

This coupled molecular and physiological simulation can be summarised as a mutual inhibition feedback between RpoS and growth rate^24^ (Fig. 2f). Using a coarse-grained exploration of the parameter space we found parameters for the stochastic simulation and Hill functions which reproduced the *WT* and *rpoS*-knockout experimental growth distributions (Fig. 2i, Supplementary Table 1) as well as the population growth rate. With these parameters set, the model then produced a long-tailed distribution of RpoS pulse heights, which decreased in prominence when the negative RpoS feedback on growth rate was removed in silico (Fig. 2g). The model also captured the rich single-cell RpoS and growth dynamics observed (Fig. 2j), as well as the anti-correlation between growth rate and RpoS (Fig. 2h).

Finally, the model correctly captured the effect of positive regulation of growth by a molecular species. Increasing *γ* concentration caused an increase in growth rate, which manifested as a positive cross-correlation between *γ* and growth rate (Supplementary Figure 7c). This corresponded well to the cross-correlation of σ^70^ and growth rate (Supplementary Figure 7a).

### Experimental RpoS and growth rate perturbations validate model

**Fig. 3:**
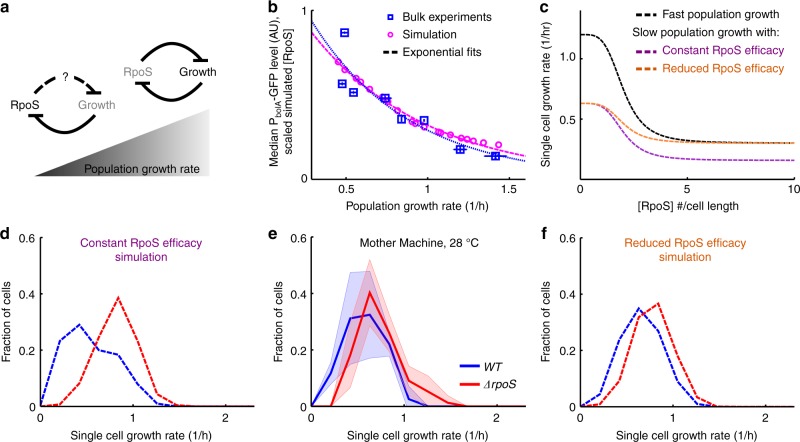
RpoS levels increase, but are less potent, at reduced population growth rate. **a** Schematic illustrating effect of reduced population growth rate. RpoS is concentrated due to lower dilution by growth rate. However, its effect on growth rate could diminish at low population growth rate. **b** Median P_bolA_-GFP (RpoS) levels in liquid culture (±std dev smaller than data point square, mean growth rate ± std dev, at least two biological replicates, see Supplementary Table 3 for details) and scaled RpoS concentration from simulations as functions of population growth rate. Dashed lines are exponential fits. Scaling factor (0.32) was found by minimising root-mean-square error between the fits over the range of observed growth rates ± 20% (0.29 to 1.6/h). **c** Hill functions of growth rate as functions of RpoS concentration used in simulations. Fast population growth corresponds to simulation matching experimentally observed growth rate at 37 °C (Fig. 2e, i). The constant and reduced efficacy models behave differently in the large RpoS concentration limit as population growth rate is reduced. **d**–**f** Growth rate histograms for *WT* and *ΔrpoS*. **e** Cells grown at reduced temperature, 28 °C, in the Mother Machine (mean ± std dev, *WT*, 4 technical replicates drawn from three biological replicates, 77 mother cells; *ΔrpoS*, 4 tech. reps. drawn from 2 bio. rep., 77 mother cells). Simulation results at corresponding population growth rate with constant RpoS efficacy (**d**) and reduced RpoS efficacy (**f**) (100 simulations for 19 values of *g*_max_, sampled every 24 h, in the final 250 h of 500 h simulations)

We tested our understanding of the feedback model by perturbing population growth rate and by overexpressing RpoS. Our model predicts overexpression of RpoS will reduce growth rate, indeed this is what we observed^39^ (see Methods section and Supplementary Figure 8). As population growth rate is reduced, RpoS levels should increase due to decreased dilution (Fig. 3a). We reduced population growth rate by reducing culture temperature, using reduced quality media, or combinations of the two (Supplementary Table 2) and imaged single cells from bulk cultures (see Methods section). Indeed, P_bolA_-GFP levels (as a proxy for RpoS levels) increased with decreasing population growth rate (Fig. 3b).

The ability of RpoS to repress growth could decrease with population growth rate due to globally reduced rates of transcription^40,41^. On the other hand, RpoS efficacy could remain constant, or even increase, allowing RpoS to control a greater portion of transcription and so repress growth more effectively. We used the model to distinguish between these possibilities. We modelled a reduction in population growth rate by decreasing *g*_max_ (see Methods section). The effect of RpoS on growth rate could scale with this maximum growth rate, reflecting a constant RpoS efficacy, or remain fixed, reflecting an attenuated RpoS efficacy. We modelled the former by keeping *f* constant in the RpoS Hill function as *g*_max_ was varied. The latter was done by keeping the product *f∙g*_max_ constant, thereby flattening the repressive Hill function (Fig. 3c, Supplementary Figure 9, and Methods section).

Comparing the theory to experiments, we found RpoS efficacy reduced with population growth rate, i.e., RpoS was less able to repress growth at low population growth rates. Using the Mother Machine assay and reduced culture temperatures we experimentally observed that the growth rate distributions of *WT* and *ΔrpoS* populations do not diverge (Fig. 3e, Supplementary Figure 9b). We found that the constant efficacy model overestimated the effect of RpoS on single-cell growth rate as population growth rate was reduced (Fig. 3d, e Supplementary Figure 9a, b), whereas the reduced RpoS efficacy model faithfully represented experiment (Fig. 3e, f, Supplementary Figure 9b, c). Additionally, the reduced efficacy model captured the increasing levels of RpoS at reduced population growth rates (Fig. 3b).

### (p)ppGpp does not abolish heterogeneous RpoS expression

Classically, the small molecule alarmone (p)ppGpp, has been found to affect RpoS expression levels^42,43^. We investigated the effect of (p)ppGpp on RpoS dynamics using the single-cell bulk culture assay (Supplementary Figure 10a, Methods). The primary synthase of (p)ppGpp is RelA^44,45^. We found a *ΔrelA* mutant to have similar RpoS heterogeneity to *WT* (Supplementary Figure 10b, c). However, the primary hydrolase of (p)ppGpp, SpoT, also has residual synthetic activity^45^, so we used the double mutant, *ΔrelAΔspoT*, to test cells devoid of (p)ppGpp. To support the growth of the sensitive double mutant we used supplemented minimal media (Methods)^46^. This caused bulk culture growth rates to increase to ~1.6/h from the standard growth rate of ~1.4/h, reducing RpoS expression in all strains, as expected (Supplementary Figure 10d, e, Fig. 3b, and Supplementary Table 2). We thus cultured cells at a reduced temperature of 28 °C causing growth rates to decrease to ~0.6/h, restoring mean RpoS expression (Supplementary Figure 10f, g). In this condition, we found the mean RpoS expression to be reduced slightly in *ΔrelA*, and markedly in *ΔrelAΔspoT* (Supplementary Figure 10f, g). The double mutant did not reduce the mean RpoS expression to that of *ΔrpoS*, neither was RpoS heterogeneity abolished, suggesting the RpoS dynamics do not arise solely from (p)ppGpp dynamics (Supplementary Figure 10f).

### Function of heterogeneous RpoS and noisy growth

**Fig. 4:**
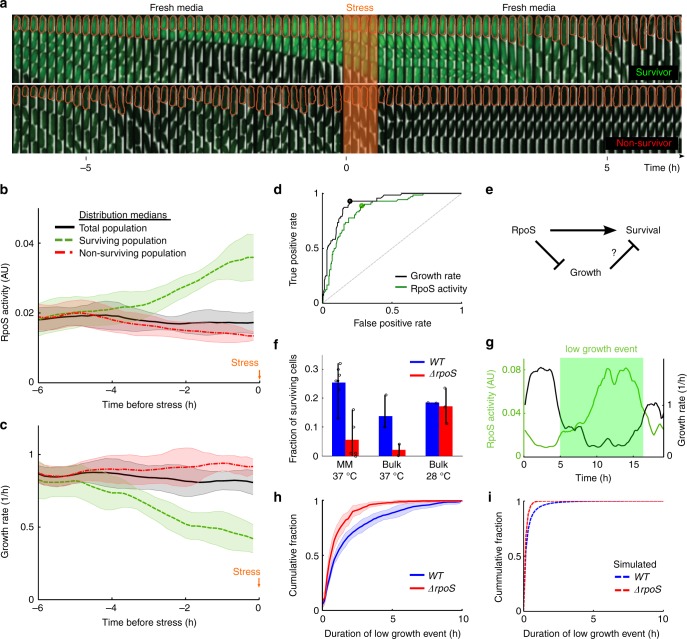
RpoS enables survival of stress by prolonging duration of slow growing state. **a** Schematic of the stress assay and sample montages of surviving (top) and non-surviving (bottom) mother cell (outlined in orange); 1 frame/10 min; phase contrast and fluorescence channel ranges identical for both montages and chosen for display. Cells were grown in fresh media, followed by application of H_2_O_2_ stress, and fresh media once again. **b** Median value of RpoS activity distributions for time points prior to stress application (*t* = 0), sorted according to survival (line and shaded area are mean ± std dev, 7 technical replicates drawn from 4 biological replicates; 72 surviving cells, 212 non-surviving, 284 total mother cells). **c** Same as **b** for growth rate. **d** Receiver operating characteristic curve for growth rate (black) and RpoS activity (green) from time point preceding stress application. Grey dashed line is true positive rate =  false positive rate. Circles represent locations of optimal thresholds (0.71/h for growth rate, 0.020 AU for RpoS activity). Area under the curve (AUC) is 0.91 for growth rate and 0.85 for RpoS activity. **e** Schematic illustrating alternative mechanisms of stress survival. High RpoS activity could directly allow cells to survive or it might first reduce growth rate, which in turn allows survival. **f** Fraction of cells surviving stress in the Mother Machine assay (mean ± max/min, *WT*: 7 tech. reps., represented as circles, drawn from 4 bio. reps., 1,087 cells, *ΔrpoS*: 5 tech. reps. drawn from 3 bio. reps., 996 cells) and bulk culture assay at two temperatures (mean ± max/min; at least two biological replicates for bulk assays, represented as circles). **g** Illustration of a low growth event based on the ROC curve optimal threshold (0.71/h) (**d**). **h** Cumulative distribution of duration of low growth events in *WT* and *ΔrpoS* populations (mean ± std dev, *WT*, 11 tech. reps. drawn from 7 bio. reps., 507 mother cells, 961 events; *ΔrpoS*, 10 tech. reps. drawn from 6 bio. rep., 274 mother cells, 484 events). **i** Same as **h** from simulations (1000 simulations run for 500 h, only the final 250 h were used; *WT*, 96,996 events and *ΔrpoS*, 69,992 events)

The RpoS regulon allows cells to survive a variety of environmental stresses, for instance oxidative stress^26,30,47^. To test the function of heterogeneous RpoS expression, we assayed the survival of exponential phase cells against hydrogen peroxide (H_2_O_2_). We used a short, intense pulse of stress to study the effect of RpoS already present in the bacteria, as opposed to the well-studied stress-induced RpoS response^30^. Using the Mother Machine we allowed cells to grow in fresh media, briefly switched to media containing H_2_O_2_, and then back to fresh media (Fig. 4a, see Methods section for details). The population of cells that survived the stress had upregulated RpoS approximately 3 h prior to the stress (Fig. 4b). Consistent with literature^30^, *rpoS* knockout populations had a reduced survival fraction compared to *WT* (Fig. 4f).

Intriguingly, the surviving population also had reduced growth rate prior to the stress (Fig. 4c). A receiver operating characteristic (ROC) curve measures how well a binary classifier performs as the threshold of the classifier is varied (see Methods section for details). Using the ROC curve, we found that both RpoS activity and growth rate immediately preceding stress application are strong predictors of survival (Fig. 4d and Methods section). This suggested two alternative hypotheses; either RpoS directly causes the survival phenotype, or it acts by first reducing growth rate, which in turn allows cells to survive the stress (Fig. 4e).

To distinguish between the two hypotheses, we noted the fraction of cells growing slower than the optimal threshold for survival increased for both *WT* and *ΔrpoS* populations as population growth rate decreased (Fig. 4d, Methods section, Supplementary Figure 9b). If RpoS directly caused survival, then the difference in survival fraction between *WT* and *ΔrpoS* populations should increase at reduced temperature due to the increased RpoS present in *WT* cells (Fig. 3b). On the other hand, if growth rate was causing survival, the difference should decrease (Fig. 3e). We tested this experimentally by a bulk culture colony forming units (CFU) stress assay (see Methods section) and found the latter (Fig. 4f). Furthermore, we observed *rpoS*-knockout cells that survived in the Mother Machine assay at 37 °C also down-regulated growth prior to stress (Supplementary Figure 11). This prompted the question: What is the role of RpoS at fast population growth rates?

To answer this question we analysed periods when cells were growing slower than the optimal threshold for survival (Fig. 4g). The role of RpoS is to prolong the duration of these slow growth events. We observed this as a higher frequency of long duration slow growth events in *WT* compared to *ΔrpoS* (Fig. 4h, Supplementary Figure 6f). The frequency with which cells attempt to grow slowly for any duration is similar for *WT* and *ΔrpoS* populations (Supplementary Figures 12a, 6g). The RpoS-growth feedback model captures this dynamic RpoS phenotype (Fig. 4i, Supplementary Figure 12b).

## Discussion

In this work, we found an instance of noisy gene expression and noisy growth coupling to produce a functional phenotype. We began with an examination of single cells drawn from bulk cultures to demonstrate the heterogeneous expression of RpoS. To unravel how this heterogeneity arose we tracked single cells over multiple generations. We found RpoS pulsing, coupled to noisy growth, generated the heterogeneity. We examined this notion further with a mathematical model of mutual inhibition between RpoS and growth, revealing how a simple feedback loop can generate the complex phenotype we observed. Finally, we showed that *E*. *coli* use their stress response system in this dynamic way to prepare for and survive sudden stressful events.

Our model of the RpoS-growth circuit simulated single cells, each growing according to stochastic molecular reactions happening inside them. This approach allowed us to compare theory directly to experiment at the single cell level as well as with summary statistics (Figs. 2, 3). Despite the predictive power of our model, we note that it is a toy model. The utility of the model is to show that RpoS can pulse given a simple mutual inhibition feedback with growth rate. However, our model suffers from a large number of lumped parameters—constants that encompass many molecular details in single numbers and so become difficult to interpret. Such a phenomenological approach can still be powerful, and has been used to reveal the bacterial division rule^48^, how noise propagates from gene expression to growth rate^23^, and the way in which gene expression can generate bistable growth^24^. To give more meaning to the parameters, it will be critical in the future to develop a more detailed model of the system. Both molecular and physiological details promise new insights. The number of adjustable parameters might also be reduced by incorporating recently developed bacterial growth laws based on proteome partitions that have been used to make remarkably accurate predictions of bulk culture phenotypes^41,49,50^.

Despite the lumped nature of the parameters, our simple model makes experimentally testable predictions about the underlying molecular details. We found *γ*, the molecule promoting growth, degraded rapidly and was present in small molecule numbers in the cell. This produced noisy growth with a growth rate distribution commensurate with experiment (Fig. 2e, i). Furthermore, growth rate and *γ* were positively correlated (Supplementary Figure 7c). These facts suggest *γ* is a transiently expressed molecule regulated by σ^70^. Alternatively, *γ* might represent the difference in abundance of two molecular species that regulate growth. Further work may reveal the existence and identity of this molecule.

Another molecular mechanism we can speculate on is how RpoS represses growth. In our model we lumped the repression into a negative Hill function (Figure 2f). σ^70^ promotes growth when bound to the RNA polymerase core. If RpoS could supplant σ^70^ it might reduce growth^51,52^. However, σ^70^ binds the RNAP core with much greater affinity^53^. By upregulating a σ^70^ sequestering anti-sigma factor, Rsd^54,55^, RpoS may effectively compete with σ^70^. Incorporating such molecular details in future work may shed light on how sigma factor competition affects RpoS heterogeneity and growth dynamics.

The regulon of *rpoS* is well studied, allowing us to speculate on the molecular mechanism underlying the survival phenotype. *E*. *coli* has two catalase genes, *katE* and *katG*, encoding hydroperoxidase (HP) I and II, respectively^56^, and alkyl hydroperoxide reductase genes, *ahpCF*^57^. While *katE* expression is upregulated by RpoS, *katG* and *ahpCF* are expressed independently of RpoS^58^. Survival against H_2_O_2_ attack (Fig. 4) in the *WT* is likely a combination of direct induction of KatE and accumulation of KatG and AhpCF due to slow growth, whereas *ΔrpoS* survive due to only the latter effect (Supplementary Figure 11).

The connection between our dynamic RpoS phenomenon and that of persistence needs further investigation. Both high (p)ppGpp concentration^59,60^ and slow growth^61,62^, independent of (p)ppGpp, have been implicated in the production of persister cells. Persistence is a stochastic phenotype that allows a small fraction of cells, orders of magnitude more rare than the RpoS phenomenon we report here, to survive antibiotic treatment^2,3^. Our data show that, while not essential, (p)ppGpp is important in the control of RpoS dynamics (Supplementary Figure 10). Indeed, RpoS has also been implicated in persister formation^60^, although this connection might be growth condition dependent. For example, others have found an absence of high-RpoS expressing cells when using diluted rich media^63^. Exposure to antibiotics can enhance subsequent survival against acid stress, a response mediated by RpoS^64^. It may be that persisters are an extreme case of the high-RpoS, slow-growth state we have revealed. To untangle the connection between the dynamics of (p)ppGpp and RpoS, an in vivo reporter for (p)ppGpp concentration that is independent of RpoS may be useful, although likely challenging to produce.

We have shown bacteria can modulate their growth using heterogeneous gene expression in anticipation of an adverse environment and have produced a simple model to explain our observations. The coupling that we have uncovered allows populations to balance the cost of RpoS expression for an individual cell against the protective benefit gained by the population, using the whole population growth rate. In the future, it will be interesting to investigate whether this balance can be modulated in lab evolution experiments under varying environmental conditions.

## Methods

### Strains and media

See Supplementary Table 3 for list of strains. M9 (1xM9 Salts, 2 mM MgSO_4_, 0.1 mM CaCl_2_; 5xM9 Salts 34 g/L Na_2_HPO_4_, 15 g/L KH_2_PO_4_, 2.5 g/L NaCl, 5 g/L NH_4_Cl) supplemented with 0.2% Casamino acids and 0.4% glucose as carbon source. Media for Mother Machine experiments was also supplemented with 0.2 mg/mL Bovine Serum Albumin (BSA). For growth rate perturbation experiments glucose was replaced with 0.4% mannose and Casamino acids with 1 mM thiamine (see Supplementary Table 2 for further details). For RpoS overexpression experiments, cells were grown in LB with 125 μg/mL Carbenicillin. For transcriptional reporters, 25 μg/mL Kanamycin or 100 μg/mL Spectinomycin was used as appropriate. In (p)ppGpp experiments with media denoted ‘M9 Supp’, Casamino acids were replaced with 400 μg/mL of serine, and 40 μg/mL of the remaining 19 amino acids, and the media was supplemented with 2 μM FeS0_4_, and 1 μg/mL thiamine^46,59^.

### Reporter plasmid

Reporter plasmids were sourced from the Alon library^35^ using standard procedures and Qiagen Miniprep kits. The plasmid antibiotic resistance was changed from Kan^r^ to Spec^r^, where specified, by PCR amplifying the Spec^r^ from the pDR111 plasmid (kind gift of Prof. Rudner, Supplementary Table 4), then using restriction digestion of the original reporter and ligation assembly. Strains were transformed with the appropriate reporter plasmids by using a variant of the Top10 Chemical Competence protocol (OpenWetWare) followed by standard transformation by heat shock. Either an overnight culture or cells taken directly from glycerol stocks were grown up to exponential phase in LB. The cells were washed and concentrated in pre-chilled CCMB80 buffer 2–3 times (CCMB80: 10 mM KOAc, 80 mM CaCl_2_∙2H_2_0, 20 mM MnCl_2_∙4H_2_O, 10 mM MgCl_2_∙6H_2_O, 10% glycerol). Next the plasmid was added to the cells and the mixture incubated on ice for 20–30 min. After a 1 min 42 °C heat shock, cells were allowed to recover in 1 mL LB at 37 °C for 1 h before plating on LB agar plates with appropriate selection overnight.

### Knockout construction and chromosomal integration of reporter

Knockout strains were sourced from the Keio collection^36^. The knockout site with Kan^r^ was amplified by PCR and used to perform knockouts in the MG1655 *E*. *coli* strain (see Supplementary Tables 3 and 4). Knockouts were carried out by the commercial Red/ET Recombination system (Gene Bridges, Germany) following the recommended protocol. However, instead of electroporation for transforming with the Red/ET recombination plasmid and FLPe flipase plasmid we used chemical transformation. The transformation was as above except the recovery was carried out at 30 °C and 1000 rpm in a benchtop shaker and plates incubated at 30 °C as the plasmids’ replication ceases at 37 °C. Knockouts were verified by colony PCR and sequencing. Knockins were performed similarly to knockouts with the Red/ET recombination system (Gene Bridges). The integrated DNA was amplified off the reporter plasmid and inserted between the *nupG* and *speC* genes (see Supplementary Table 4). The reporter plasmids were sequenced and used as references for the integration.

### Single cell bulk culture snapshots

We used the imaging protocol developed previously^32^ with minor modifications. Cells were grown from glycerol stocks or from colonies on LB agar plates streaked with the glycerol stocks in M9 at 37 °C to late exponential phase and then diluted back into M9 to an OD of 0.01 (Optical Density, 600 nm; Ultrospec 10, Amersham Biosciences, UK). After re-growing for approximately 2 h 20 min, up to early exponential phase (OD~0.2), 0.3 μL of the cell culture was spotted onto pads of 1.5% low-melting agarose in Phosphate-Buffered Saline (PBS). Cells were imaged expediently, typically within ~20 min of leaving the incubator.

### Bulk culture snapshots of fixed cells

Cells were grown as above. After measuring the OD of the culture, 37% formaldehyde in methanol was added to the remaining culture for a final concentration of ~3.7% formaldehyde. Formaldehyde was added within 6 min of leaving the shaking incubator, and left to mix for ~27 min. Cells were then spun at 4500×*g* for 5 min. The pellets were washed in PBS twice with the same spinning procedure, and finally resuspended in 100 μL PBS. A 0.3 μL droplet was then spotted on agarose pads as above and imaged.

### Population growth rate perturbation

Cells were grown from glycerol stocks using the modified media and temperature into exponential phase. Optical density measurements were taken after cells were diluted and grown up to exponential phase for imaging.

### RpoS over-expression

Cells were streaked on LB agar plates and colonies picked into 2 mL LB. Cultures were grown in LB at 37 °C and diluted to OD 0.01 into 10 mL LB supplemented with either IPTG or water and grown again. OD measurements were taken at intervals and cells imaged as above.

### Mother Machine microfluidic device

The Mother Machine microfluidics device has been developed previously^33^. It consists of a feed trench (~50 μM × 100 μM × 30 mm) with many channels (~1.4 μM × 1.4 μM × 25 μM) attached perpendicular to the trench. These channels hold the cells and media is supplied to the cells via the trench. We used an epoxy mould to fabricate our devices, which was a kind gift of Professor Suckjoon Jun. The devices were fabricated by casting Sylgard 184 polydimethylsiloxane (PDMS) (Dow Corning, USA) with a ratio of 10:1 base to curing agent onto the mould and cured overnight at 65 °C. The chips were then cut out and plasma bonded (Femto Plasma System, Diener, Germany) to a glass bottom dish (HBSt-5040, Wilco Wells, Netherlands). To strengthen the bonding the chips were incubated for approximately 10 min at 65 °C. The chips were passivated with 20 mg/mL Bovine Serum Albumin (BSA) for approximately one hour at 37 °C prior to cell loading.

### Mother Machine movies data acquisition

Cells were grown from glycerol stocks as above. They were concentrated by centrifugation (~1500×*g* for 10 min) and injected into the Mother Machine devices. A second centrifugation step for 5 min at 4000 rpm using a spin coater (Polos Spin150i, SPS, Netherlands) forced cells into the channels. Cells were allowed to settle in the device while being supplied with fresh media for ~2 h prior to beginning image acquisition. Media was supplied at a flowrate of 1 ml/h by either a Fluigent pressure pump (MFCS-EZ, Fluigent, France) with an M- Flow sensor (Fluigent, France) or a syringe pump (Fusion 100, Chemyx, USA).

### Agarose pad movies

The agarose pad movie protocol has been developed previously^32^. 1.5% low-melting agarose was melted in M9, allowed to cool, supplemented with antibiotics, and cast sandwiched between two coverslips. Cells were grown from glycerol stock overnight in M9 media, diluted to an OD of 0.01 and grown up to exponential phase. The culture was diluted and spotted onto pads cut out from the cast agarose. The cells were incubated at 37 °C during the movie.

### CellASIC movies

We loaded exponential phase cells prepared as in the agarose pad movies into the CellASIC ONIX B04A-03 microfluidic device using the manufacturer’s protocol (EMD Millipore Corporation). The cells were supplied with M9 and incubated at 37 °C during the movie.

### Microscopy

We used a widefield microscope with epifluorescence and phase contrast imaging modes (Nikon Ti-eclipse, Nikon, UK) equipped with the Nikon Perfect Focus (PFS) Unit. Illumination for the epifluorescence was provided by a white light LED source (SOLA SE Light Engine or Spectra X Light Engine, Lumencor, USA), transmitted by a liquid light guide (Lumencor, USA), through a fluorescence filter cube (GFP Channel: 49002-ET-EGFP, excitation: ET470/40×, dichroic: T495LP, emitter: ET525/50 m; RFP Channel: 41027-Calcium Crimson, excitation: HQ580/20×, dichroic: Q595LP, Emitter: HQ630/60 m, Chroma, USA), and a CFI Plan Apochromat 100× oil immersion objective (NA 1.45, Nikon). Phase contrast illumination was provided by a 100 W lamp via a condenser unit (Nikon). Images were acquired on a CoolSNAP HQ^2^ camera (Photometrics, USA). The sample was held in motorised stages (Nikon). The sample was incubated along with much of the microscope body using a temperature controlled, heated chamber (Solent Scientific, UK). The microscope was controlled with MetaMorph software (version 7.8.10.0, Molecular Devices, USA). Fluorescent beads (TetraSpeck microspheres, 0.5 μM, Molecular Probes, USA) were imaged as a calibration standard.

### Bulk culture growth rate and single-cell gene expression

A custom MATLAB (Mathworks, USA) script based on the published Schnitzcells software was used for image analysis^32^. The microscope was calibrated for each experiment with fluorescent beads to mitigate the effect of non-uniform sample illumination and daily variations in the apparatus. Cells were taken from a field of view computed from the beads to be within 80% of maximum intensity. Cells were segmented in the phase contrast channel. The mean fluorescence was then the corresponding pixels in the GFP channel normalised to cell area. A threshold was applied to exclude debris and substrate autofluorescence was subtracted from the mean cell fluorescence. Finally, the cell fluorescence was normalised by the fluorescence of the top 2% of fluorescent beads, which were also corrected for substrate autofluorescence. For growth perturbation experiments growth rate was calculated by fitting an exponential curve to the OD measurements. Growth rate was not computed for RpoS over expression experiments due to the non-monotonic nature of the change in culture density.

### Single-cell growth rates from Mother Machine movies

Cell segmentation was done on the phase contrast channel using MATLAB (Mathworks, USA) scripts. The mother cell—the cell that remained at the end of growth channels farthest from the feed trench—was isolated and tracked. The automated image analysis was robust, however it occasionally produced artefacts. Thus, every frame from the automated segmentation used in subsequent analysis was manually checked, and corrected if necessary. Cells that did not grow for the entire duration of the movie were discarded in this process. We numerically computed the relative growth rate, *g* = *l*^−1^d*l/*d*t*, at each frame, where *l* is cell length. Throughout the manuscript we refer to this relative growth rate of single cells simply as growth rate. We first computed the numerical derivative of cell length as the difference in cell length between consecutive frames (Δ*t* = 10 min), d*l/*d*t ~ (l*_*t+1*_ *− l*_*t*_*)/*Δ*t*. Despite the manual image curation, unphysical, negative growth rates occasionally resulted due to segmentation artefacts. These were corrected by replacing the negative values with the mean of the nearest frames with non-negative values. The numerical derivative was normalised by the initial length, *l*_*t*_, *g ~ (l*_*t+1*_ *− l*_*t*_*)/(Δt∙l*_*t*_*)*, and then smoothed with a moving average filter spanning five frames (Supplementary Figure 4a, b). The growth rate sample traces in Fig. 2 and Supplementary Figures 1, 4, and 6 were smoothed again with a moving average filter spanning five frames for display. The population growth rate of mother cells (used in Fig. 3 and Supplementary Figure 9, as well as for comparison in the coarse grain parameter search for simulations) was computed as *g*_pop_ *=* *ln(2)/t*_D_, where *t*_D_ was found by numerically solving:

1 $$\frac{{P_{{\mathrm{final}}}}}{{P_{{\mathrm{initial}}}}} = 2 = \mathop {\sum }\limits_i n_i2^{t_{\mathrm{D}}/c_i},$$

where *P*_*x*_ are number of cells, *n*_*i*_ are the fraction of cells growing with cell cycle time *c*_*i*_. We note that the Mother Machine technique over-represents slow growing cells compared to bulk culture since the slow growing cells do not have to compete with fast cells in the Mother Machine.

### Single-cell promoter (RpoS) activity from Mother Machine movies

Gene expression level was calculated as above for bulk single cell analysis. Calibration to beads was done using only the top 2% normalisation—no cells were excluded due to position in the field of view. Promoter activity (*A*) is defined as the normalised rate of production of the gene under the control of a promoter ($\tilde A$)^21,32^:

2 $$\frac{{{\mathrm{d}}F}}{{{\mathrm{d}}t}} = \tilde A - p \cdot F,$$

where *F* is the gene product, GFP in the case of the reporters used here, and *p* is a constant accounting for degradation and bleaching. If the promoter is RpoS sensitive, then *A* is RpoS activity, and if σ^70^ sensitive, σ^70^ activity. To extract promoter activity from the observables of cell length (*l)* and mean fluorescence (*M*) per cell we note total fluorescence is the product of mean fluorescence and cell volume (*V*):

3 $$F = M \cdot V$$

By the product rule:

4 $$\frac{{{\mathrm{d}}M}}{{{\mathrm{d}}t}} \cdot V + M \cdot \frac{{{\mathrm{d}}V}}{{{\mathrm{d}}t}} = \tilde A - p \cdot M \cdot V,$$

We assume the diameter of the cell remains constant, reducing the above to:

5 $$\frac{{{\mathrm{d}}M}}{{{\mathrm{d}}t}} \cdot l + M \cdot \frac{{{\mathrm{d}}l}}{{{\mathrm{d}}t}} = \tilde A - p \cdot M \cdot l,$$

where constants are absorbed in $\tilde A$. Finally, rearranging we obtain promoter activity as the component of the time-derivative of the mean fluorescence corrected for relative growth rate and bleaching, and normalised by cell volume (length)^21,32^ (see Supplementary Figure 4):

6 $$A = M\left( {\frac{1}{l}\frac{{{\mathrm{d}}l}}{{{\mathrm{d}}t}} + p} \right) + \frac{{{\mathrm{d}}M}}{{{\mathrm{d}}t}},$$

We computed promoter activity numerically. The relative growth rate, *g* = *l*^−1^d*l/*d*t*, was calculated as above. The mean fluorescence, *M*, was smoothed with a moving average filter spanning five frames, and then *dM/*d*t* was calculated by taking the numerical derivative of the smoothed mean fluorescence (Supplementary Figure 4c, d). The promoter activity sample traces in Fig. 2 and Supplementary Figures 4 and 6 were smoothed again with a moving average filter spanning five frames for display. We set *p* = 0.1. Our conclusions were not sensitive to the value of *p* selected. RpoS activity peaks (Fig. 2c) were found by first smoothing promoter activity with a moving average filter spanning five frames and then using the built-in MATLAB function findpeaks to identify local maxima. For each of these maxima, the highest value of the un-smoothed promoter activity within a window of seven frames centred on each local maximum was identified as the peak.

### Cross-correlation of growth rate and promoter activity

The normalised cross-correlation between growth rate and promoter activity was computed as follows:

7 $$\tilde c_{g - A}({\mathrm{\Delta }}t) = \mathop {\sum }\limits_{t \in \,{\mathrm{all}}\,{\mathrm{time}}} \frac{{\left( {g\left( {t + {\mathrm{\Delta }}t} \right) - \bar g} \right)}}{{\sqrt {c_{g - g}(0)} }}\frac{{\left( {A\left( t \right) - \bar A} \right)}}{{\sqrt {c_{A - A}(0)} }},$$

where *g* is growth rate, *A* is promoter activity, Δ*t* is the time difference between the two signals, overbars indicate averages over time, and *c* is the auto-correlation:

8 $$c_{x - x}({\mathrm{\Delta }}t) = \mathop {\sum }\limits_{t \in \,{\mathrm{all}}\,{\mathrm{time}}} \left( {x\left( {t + {\mathrm{\Delta }}t} \right) - \bar x} \right)\left( {x\left( t \right) - \bar x} \right),$$

where *x* is either promoter activity or growth rate. The cross-correlation was implemented using the built-in MATLAB function xcov with the “coeff” option.

### Bulk culture CFU assay

Cells were grown into exponential phase from glycerol stocks at either 37 or 28 °C and diluted into 10 mL fresh media. They were grown into exponential phase again and aliquoted into 2 mL cultures. These aliquots were exposed to either water or 26 mM H_2_O_2_ and incubated for a further 20 min. Cultures were then serially diluted in M9 and plated on LB agar plates. The colonies on the plates were counted after an overnight incubation at 37 °C to determine the CFU. Survival fraction was computed as cells/mL from the stress condition divided by the cells/mL from the water condition. Averages were taken over all plates that were in the dynamic range of the assay (30–300 colonies per plate).

### Mother Machine survival assay

Cells were loaded into the Mother Machine as above. Cells were allowed to grow in fresh media for 10 h, then exposed to 35 mM H_2_O_2_ for 35 min and then supplied with fresh media again for at least 12 h. The media was switched with a Fluigent 2-switch or M-switch (Fluigent, France). Two 35 min pulses of 3 to 12 mM propidium iodide were supplied with the second round of fresh media and the cells were imaged in the RFP channel to observe DNA chelation of dead cells. This approach was not robust for identifying survivors and dead cells. Thus the movies for each mother cell were manually curated to determine survival using solely the phase contrast channel. If the cell began growing post-H_2_O_2_ treatment and before the movie ended, it was counted as a survivor. Ambiguous cases were excluded from the tally (*WT*, 14% of cells excluded, *ΔrpoS*, 5%), however including these cells in the survival fraction calculation did not change the results.

### ROC curve

A receiver operating characteristic (ROC) curve measures how well a binary classifier performs as the threshold of the classifier is varied. We used growth rate and RpoS activity to classify the survival of cells in the Mother Machine survival assay. The true positive rate (TPR) as a function of the threshold was computed as:

9 $${\mathrm{TPR(threshold)}} = \frac{{{\mathrm{\# }}\,{\mathrm{Surviving}}\,{\mathrm{cells}}\,{\mathrm{past}}\,{\mathrm{threshold}}}}{{{\mathrm{Total}}\,{\mathrm{\# }}\,{\mathrm{surviving}}\,{\mathrm{cells}}}}$$

Similarly, the false positive rate (FPR) was computed as:

10 $${\mathrm{FPR(threshold)}} = \frac{{{\mathrm{\# }}\,{\mathrm{Non \operatorname{-} surviving}}\,{\mathrm{cells}}\,{\mathrm{past}}\,{\mathrm{threshold}}}}{{{\mathrm{Total}}\,{\mathrm{\# }}\,{\mathrm{non\operatorname{-}surviving}}\,{\mathrm{cells}}}}$$

When growth rate was used as the classifier, cells passed the threshold if their growth rate was below the tested value; while for RpoS activity if it was above. The TPR was plotted against the FPR to generate the ROC curve. The optimal threshold was computed by finding the threshold that resulted in the maximum difference between the TPR and FPR. The area under the curve (AUC), computed by numerical integration of the ROC curve, is a measure of the quality of the classifier. A perfect classifier has AUC = 1, while one that is no better than random guessing has AUC = 0.5.

### Stochastic molecular simulation coupled to single-cell growth model

We modelled a single cell growing as a function of molecular reactions occurring inside it. A single lineage was followed, *i*.*e*. only one daughter cell was followed at each cell division. To model growth, we assumed rod-shaped cells with fixed radius and modelled growing cells by the changing length at a fixed, deterministic time interval, Δ*t*:

11 $${\mathrm{\Delta }}l_i = g_{i - 1} \cdot {\mathrm{\Delta }}t \cdot l_{i - 1},$$

where *g*_*i*_ and *l*_*i*_ are the growth rate and cell length at the *i*th time point, respectively. Cell division was assumed to follow the adder rule^48^:

12 $$l_i = \left\{ {\begin{array}{*{20}{l}} {l_{i - 1} + {\mathrm{\Delta }}l_i,\mathop {\sum }\limits_{{\mathrm{last}}\,{\mathrm{division}}}^i {\mathrm{\Delta }}l_k < {\mathrm{\Delta }}L} \hfill \\ {(l_{i - 1} + {\mathrm{\Delta }}l_i)/2,\,{\mathrm{otherwise}}} \hfill \end{array}} \right.$$

where Δ*L* is a fixed length the cell must add before it can divide. The numbers of molecules in the cell were determined by a standard Gillespie stochastic simulation algorithm^14^ that ran between the deterministic steps of the growth model. Two molecular species RpoS*, r*, and growth factor, *γ*, were modelled. They were generated with zeroth order constitutive production and first order degradation reactions:

13 $$0\mathop{\longrightarrow}\limits^{{k_{rp}}}r;r\mathop{\longrightarrow}\limits^{{k_{rd}}}0;0\mathop{\longrightarrow}\limits^{{k_{\gamma p}}}\gamma ;\gamma \mathop{\longrightarrow}\limits^{{k_{\gamma d}}}0,$$

where *k*_*xp*_ are the production propensities and *k*_*xd*_ are the degradation propensities for species *x*. The reaction propensities in the Gillespie algorithm do not change with cell volume since the reactions are zeroth and first order^34^. At division the number of molecules were simply divided in half and rounded to the closest integer lower than the quotient:

14 $${\mathrm{species}}_i = \lfloor {\mathrm{species}}_{i - 1}/2\rfloor$$

The concentration of the molecular species was the number of species divided by cell length (volume):

15 $$\left[ {{\mathrm{species}}_i} \right] = \frac{{{\mathrm{species}}_i}}{{l_{i - 1}}}$$

Growth rate was a function of the concentration of the two molecular species generated most recently by the Gillespie algorithm:

16 $$g_i = g_{{\mathrm{max}}} \cdot \left( {\frac{1}{{1 + \left( {\frac{{h_\gamma }}{{[\gamma _i]}}} \right)^{n_\gamma }}}} \right) \cdot \left( {\frac{{1 - f}}{{1 + \left( {\frac{{h_r}}{{[r_i]}}} \right)^{n_r}}} + f} \right)$$

where *g*_max_ is the maximum growth rate; *f* represents the lowest growth rate can be reduced to in the limit of infinite RpoS concentration; *h*_*γ*_ and *h*_*r*_ are the values of growth factor and RpoS leading to half-maximal growth, respectively; and *n*_*γ*_, and *n*_*r*_ are the Hill coefficients. Growth factor was considered a downstream target of σ^70^ so *n*_*γ*_ was positive, while *n*_*r*_ was chosen to be negative to capture the repressive effect of RpoS on growth. Growth perturbation simulations were implemented by varying *g*_max_, while all other parameters were kept constant. However, in the reduced RpoS efficacy model the parameter *f* was increased to keep the product *f∙g*_max_ constant. See Supplementary Table 1 for parameter values used and Supplementary Note for the pseudo code of the algorithm. Traces in Figure 2j were smoothed twice with a moving average filter spanning five frames for display.

### Code availability

Code used for simulations and for analysis of data reported in this study is available upon request from the corresponding author.

## Electronic supplementary material

Supplementary Information

Description of Additional Supplementary Files

Supplementary Movie 1

Supplementary Movie 2

Supplementary Movie 3

Supplementary Movie 4

Supplementary Movie 5

Reporting Summary

## Data Availability

Single cell datasets for *WT* and *ΔrpoS* cells used in Fig. 2 are available at https://gitlab.com/slcu/teamJL/Patange_etal_2018. All additional data that support the findings reported in this study are available in this article and its Supplementary Information files, or upon request from the corresponding author.
