# Supplementary material for: Escherichia coli can survive stress by noisy growth modulation
Source: Nat Commun. 2018 Dec 17;9:5333. doi: 10.1038/s41467-018-07702-z (PMC6297224; doi:10.1038/s41467-018-07702-z)
Supplement: Supplementary file 1 — Supplementary Information [file 41467_2018_7702_MOESM1_ESM.pdf]

Supplementary Information

Escherichia coli can survive stress by noisy growth modulation

Patange *et al.*

Supplementary Tables

Supplementary Table 1: Model parameters.

Parameter	Value in model	Value in physical units	Description
		Gillespie	
$k_{\gamma p}$	2.2	13 hr^{-1}	γ zeroth order production rate constant
$k_{\gamma d}$	0.2	1.2 hr^{-1}	γ first order degradation rate constant
k_{rp}	0.3	1.8 hr^{-1}	RpoS zeroth order production rate constant
k_{rd}	0.01	0.06 hr^{-1}	RpoS first order degradation rate constant
γ_{init}	11	11 molecules	Initial value of γ
r_{init}	1	1 molecule	Initial value of RpoS
		Growth	
ΔL	1	2 μm	Length cell must grow before dividing
l_i	1	2 μm	Initial cell length
		Coupling growth and Gillespie models	
g_{max}	1.2	7.2 hr^{-1} (0.7 hr^{-1})	Maximum (average) growth rate achievable by cell
h_γ	17	17 molecules/cell	Half-maximum value for γ-growth Hill function
n_γ	1	-	Hill coefficient for γ-growth Hill function
h_r	2	2 molecules/cell	Half-maximum value for RpoS-growth Hill function
n_r	-4	-	Hill coefficient for RpoS-growth Hill function
f	0.25	-	Minimum value RpoS can reduce growth rate by
		Technical parameters	
	100 or 1,000	-	Number of simulations
	3000	500 hrs	Number of time steps
	0.005	3 s	Simulation time resolution
	1/0.005 = 200	10 min	Sampling resolution

Supplementary Table 2: Growth conditions and population growth rates for growth perturbation experiments.

	WT		$\Delta rpoS$	
Growth condition*	Biological replicates; number of cells	Growth rate (1/hr), mean ± std dev	Biological replicates; number of cells	Growth rate (1/hr), mean ± std dev
0.4% glucose, 0.2% casamino acids (37°C)	4; 711	1.42 ± 0.07	3; 427	1.42 ± 0.08
0.4% glucose, 0.2% casamino acids (33°C)	2; 547	0.98 ± 0	2; 510	1.02 ± 0
0.4% glucose, 0.2% casamino acids (28°C)	2; 747	0.55 ± 0.02	2; 601	0.59 ± 0.01
0.4% mannose, 0.2% casamino acids (37°C)	3; 720	1.20 ± 0.04	2; 453	1.23 ± 0
0.4% mannose, 0.2% casamino acids (33°C)	2; 346	0.84 ± 0	2; 511	0.85 ± 0.02
0.4% mannose, 0.2% casamino acids (28°C)	2; 604	0.48 ± 0.02	2; 595	0.51 ± 0.01
0.4% glucose, 1 mM thiamine (37°C)	2; 896	0.74 ± 0.04	2; 536	0.74 ± 0.02
0.4% mannose, 1 mM thiamine (37°C)	3; 2,719	0.49 ± 0.02	3; 2,298	0.52 ± 0.03

*M9 supplemented with the following and grown at (temperature).

Supplementary Table 3: List of strains.

Strain name	Genotype or description	Construction procedure	Source
MG1655*	*WT* of reporter library [1]		Yale CGSC (CGSC # 6300 and 7740), and gift of Kenn Gerdes
MG1655+bolA	Same as MG1655; P_{bolA}-*GFP*, Kan^r	Reporter plasmid P_{bolA}-*GFP*, Kan^r [1] in MG1655	Reporter library [1] and this work
MG1655+blc	Same as MG1655; P_{blc}-*GFP*, Kan^r	Reporter plasmid P_{blc}-*GFP*, Kan^r [1] in MG1655	Reporter library [1] and this work
MG1655+poxB	Same as MG1655; P_{poxB}-*GFP*, Kan^r	Reporter plasmid P_{poxB}-*GFP*, Kan^r [1] in MG1655	Reporter library [1] and this work
MG1655+rpsL	Same as MG1655; P_{rpsL}-*GFP*, Kan^r	Reporter plasmid P_{rpsL}-*GFP*, Kan^r [1] in MG1655	Reporter library [1] and this work
MG1655+lacI	Same as MG1655; P_{lacI}-*GFP*, Kan^r	Reporter plasmid P_{lacI}-*GFP*, Kan^r [1] in MG1655	Reporter library [1]
MGChrPbolA	Same as MG1655 with chromosomally integrated P_{bolA}-*GFP*, Kan^r	Used Red/ET system and PCR product amplified from reporter plasmid P_{bolA}-*GFP*, Kan^r [1]	This work
DrpoS*	MG1655 with Δ*rpoS::Kanr*	Used a PCR product from Keio collection Δ*rpoS* strain [2]	This work
DrpoSF-	Same as DrpoS, markerless	FLPe recombinase	This work
DrpoSF-+bolA	Same as DrpoSF-; P_{bolA}-*GFP*, Kan^r	Reporter plasmid P_{bolA}-*GFP*, Kan^r [1] in DrpoSF-	This work
DrpoSF-+blc	Same as DrpoSF-; P_{blc}-*GFP*, Kan^r	Reporter plasmid P_{blc}-*GFP*, Kan^r [1] in DrpoSF-	This work
DrpoSF-+poxB	Same as DrpoSF-; P_{poxB}-*GFP*, Kan^r	Reporter plasmid P_{poxB}-*GFP*, Kan^r [1] in DrpoSF-	This work
DrpoSF-+rpsL	Same as DrpoSF-; P_{rpsL}-*GFP*, Kan^r	Reporter plasmid P_{rpsL}-*GFP*, Kan^r [1] in DrpoSF-	This work
DrpoSF-+lacI	Same as DrpoSF-; P_{lacI}-*GFP*, Kan^r	Reporter plasmid P_{lacI}-*GFP*, Kan^r [1] in DrpoSF-	This work
DrpoSF-ChrPbolA	Same as DrpoSF- with chromosomally integrated P_{bolA}-*GFP*, Kan^r	Used Red/ET system and PCR product amplified from reporter plasmid P_{bolA}-*GFP*, Kan^r [1]	This work

MGmCherry	Same as MG1655, with *rpoS::mCherry* chromosomally integrated, replacing *rpoS*	-	Gift of Kenn Gerdes
MGmCherry+bolA	Same as MGmCherry; P_{bolA}-*GFP*, Kan^r	Reporter plasmid P_{bolA}-*GFP*, Kan^r [1] in MGmCherry	This work
MG1655+bolASpec	Same as MG1655; P_{bolA}-*GFP*, $Spec^r$	Reporter plasmid P_{bolA}-*GFP*, Kan^r [1] in MG1655, with antibiotic switched from Kan^r to $Spec^r$	This work
DrpoSF-+bolASpec	Same as DrpoSF-; P_{bolA}-*GFP*, $Spec^r$	Reporter plasmid [1] in DrpoSF-, with antibiotic switched from Kan^r to $Spec^r$	This work
PDC47	Same as MG1655 with markerless $\Delta relA$ and Δ*spoT::cat*	-	Gift of Kenn Gerdes [3]
PDC47+bolA	Same as PDC47 with P_{bolA}-*GFP*, Kan^r	Reporter plasmid P_{bolA}-*GFP*, Kan^r [1] in PDC47	This work
BW25113	*WT* of knockout library [2]		Yale CGSC # 7636
KDr	Same as BW25113 with Δ*rpoS::*Kan^r	-	From Keio collection [2]
KDr+bolASpec	Same as KDr with P_{bolA}-*GFP*, $Spec^r$	Reporter plasmid P_{bolA}-*GFP*, Kan^r [1] in KDr, with antibiotic switched from Kan^r to $Spec^r$	This work
DrelA	Same as BW25113 with Δ*relA::*Kan^r	-	From Keio collection [2] (plates 53 and 54)
DrelA+bolA	Same as DrelA with P_{bolA}-*GFP*, $Spec^r$	Reporter plasmid P_{bolA}-*GFP*, Kan^r [1] in DrelA, with antibiotic switched from Kan^r to $Spec^r$	This work
MC4100DE3 (pRPOS)	MC4100 *WT*, expressing T7 RNA polymerase, contains IPTG-inducible RpoS construct	-	Gift of Herb Schellhorn [4]
MC4100DE3 (pRPOS)+bolA	Same as MC4100DE3 (pRPOS); P_{bolA}-*GFP*, Kan^r	Reporter plasmid P_{bolA}-*GFP*, Kan^r [1] in MC4100DE3 (pRPOS)	This work
HS1600DE3 (pRPOS)	Same as MC4100DE3 except *rpoS13::Tn10*	-	Gift of Herb Schellhorn [4]
HS1600DE3 (pRPOS)+bolA	Same as HS1600DE3 (pRPOS); P_{bolA}-*GFP*, Kan^r	Reporter plasmid P_{bolA}-*GFP*, Kan^r [1] in HS1600DE3 (pRPOS)	This work

*Isolate CGSC #6300 was used as the parent strain for Mother Machine experiments due to superior loading properties. For liquid culture experiments we used isolates CGSC #6300 and CGSC #7440, from the Stock Centre, isolates from the Reporter library [1], and an isolate that was a kind gift of Prof. Kenn Gerdes.

Supplementary Table 4: List of primers.

Primer name	Primer description	Sequence
OP007	Chromosomal Integration, Fwd	ATAAACACGTTCGTGTCCCGACAGGCA-CACAGACGGTTAGCCACTAATTA-GAGCTCTCGAACCCCAGAGT
OP008	Chromosomal Integration, Rev	GTAAGAATAAAAAAAACGGGTCAC-CTTCTGGCGACCCGTTTTTCTTTGCGC-CTGCAGGTCTGGACATTTA
OP015	*rpoS*-knockout, Fwd	TGAGACTGGCCTTTCTGACAGATGCT-TACTTACTCGCGGAACAGCGCTTCTG-TAGGCTGGAGCTGCTTCG
OP016	*rpoS*-knockout, Rev	CTTTTGCTTGAATGTTCCGTCAAGGGAT-CACGGGTAGGAGCCACCTTATGATTC-CGGGGATCCGTCGACC
CV186	Plasmid resistance change from Kan^r to $Spec^r$, Fwd	AAAGATCTGATCAAGAGACAGGATGAG-GATCGTTTCGCTTGAATACATACGAAC
CV187	Plasmid resistance change from Kan^r to $Spec^r$, Fwd	AAAGATCTAAAATAGTGAGGAG-GATATATTTG
CV188	Plasmid resistance change from Kan^r to $Spec^r$, Rev	AAAGAGCTCTTATAATTTTTTTAATCT-GTTATTTAAATAG

Supplementary Note - Model Pseudocode

```
for k = 1 to number of simulations
    %initializing first step

    for t = 1 to Number of time steps
        %first run the rpoS Gillespie:

        while accumulated Gillespie time does not exceeds growth clock
            %Perform standard Gillespie algorithm
        end

        %compute concentration of the molecules

        %Update growth rate using Hill function

        %adder rule:
        if added length > adder value
            %divide cell and molecules in half
        else
            %increase cell length
        end

        %store values with sampling resolution
        if mod(t,storestep) == 1
            %store simulation step
        end
    end

end
```

Supplementary Figures

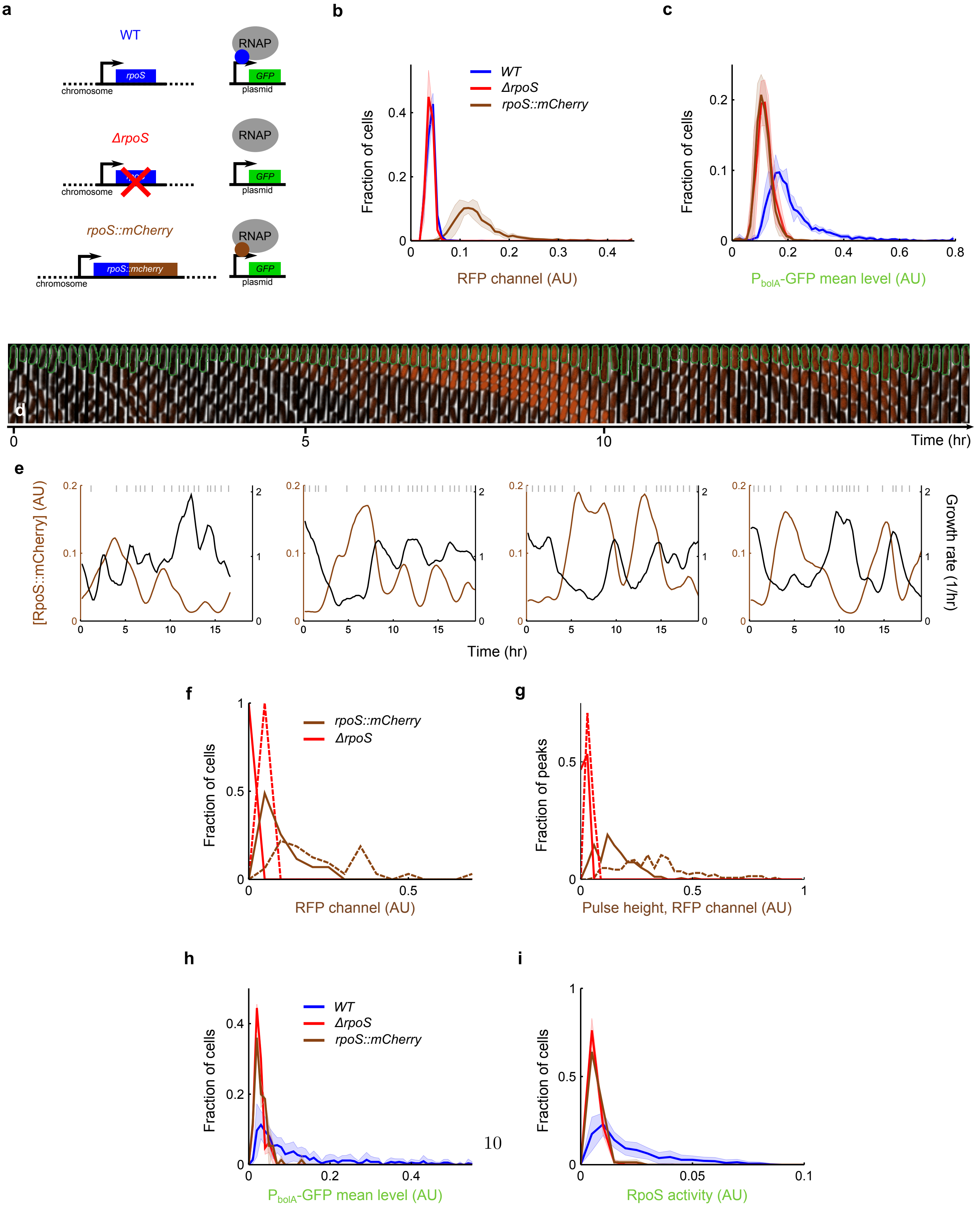

Supplementary Figure 1

(caption continued)

Supplementary Figure 1 caption, continued

Translational fusion of RpoS::mCherry is heterogeneously distributed, but is not functional. a, Schematic of the *rpoS::mCherry* strain compared to *WT* and $\Delta rpoS$ as found in remainder of the paper. b, c Measurements of cells in bulk culture. b, Histograms of RpoS::mCherry concentration measured as mean RFP channel fluorescence per cell compared to *WT* and $\Delta rpoS$ controls lacking the fluorescent construct. *WT* (5 biological replicates, 2,951 cells, mean = 0.041 AU, CV = 0.20, excluding 4 outliers), $\Delta rpoS$ (3 biological replicates, 1,525 cells, mean = 0.039 AU, CV = 0.16), and rpoS::mCherry (8 biological replicates, 4,949 cells, mean = 0.14 AU, CV = 0.40, excluding 27 outliers). c, Histograms of mean GFP fluorescence per cell *WT* (5 biological replicates, 3,065 cells, mean = 0.22 AU, CV = 0.51), $\Delta rpoS$ (3 biological replicates, 1,623 cells, mean = 0.12 AU, CV = 0.24), and *rpoS::mCherry* (8 biological replicates, 4,325 cells, mean = 0.11 AU, CV = 0.26). d-i Measurements of cells in the Mother Machine. d, Sample montage of a mother cell (green outline) in the Mother Machine pulsing on RpoS::mCherry (1 frame/10 minutes; phase contrast and fluorescence channel ranges chosen for display). e, Sample time traces of RpoS::mCherry concentration and growth rate for four mother cells illustrating pulsing. Traces smoothed with a moving average filter spanning five frames, for display. f, Histograms of RpoS::mCherry concentration measured as mean RFP channel fluorescence per cell compared to $\Delta rpoS$ control. $\Delta rpoS$ (2 biological replicates shown separately due to technical discrepancy in background; dashed line: 30 cells, mean = 0.037 AU, CV = 0.21, solid line: 33 cells, mean = 0.015 AU, CV = 0.23), and *rpoS::mCherry* (2 biological replicates shown separately; dashed line: 32 cells, mean = 0.22 AU, CV = 0.67, solid line: 43 cells, mean = 0.10 AU, CV = 0.64). g, Pulse height histograms of the same data in (f). $\Delta rpoS$ (2 biological replicates shown separately; dashed line: 202 peaks, mean = 0.040 AU, CV = 0.18, solid line: 225 peaks, mean = 0.016 AU, CV = 0.22), and *rpoS::mCherry* (2 biological replicates shown separately; dashed line: 145 peaks, mean = 0.34 AU, CV = 0.51, solid line: 179 peaks, mean = 0.15 AU, CV = 0.55). h, Mean expression from transcriptional reporter, P_{bolA}-*GFP*. *WT* (11 technical replicates drawn from 7 biological replicates, 505 cells, mean = 0.14 AU, CV = 0.98), $\Delta rpoS$ (2 biological replicates, 63 cells, mean = 0.026 AU, CV = 0.44), and *rpoS::mCherry* (2 biological replicates, 75 cells, mean = 0.030 AU, CV = 0.58). i, RpoS activity measured with the transcriptional reporter. *WT* (11 technical replicates drawn from 7 biological replicates, 505 cells, mean = 0.023 AU, CV = 0.82), $\Delta rpoS$ (2 biological replicates, 63 cells, mean = 0.0065 AU, CV = 0.32), and *rpoS::mCherry* (2 biological replicates, 75 cells, mean = 0.0073 AU, CV = 0.45). Data for (f, h, and i) were taken at one frame (59) from all movies. In (b, c, h, and i), lines and shaded region are mean ± std dev, respectively.

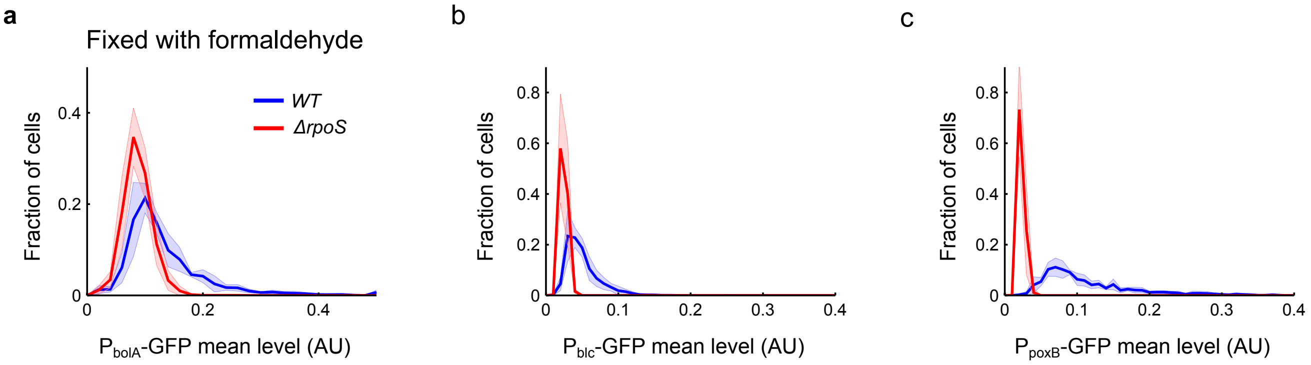

Supplementary Figure 2: Alternative imaging protocol and transcriptional reporters validate RpoS heterogeneity. a, Fixing cells with formaldehyde while still in liquid culture does not eliminate long-tailed distribution of RpoS. Transcriptional fusion of P_{bolA}-GFP in WT (6 biological replicates, 3,012 cells, mean = 0.13 AU, CV = 0.57) and $\Delta rpoS$ (6 bio. reps., 3,292 cells, mean = 0.087 AU, CV = 0.28). b, c, Alternative RpoS reporters have long-tailed distributions; the long tails vanish in the *rpoS*-knockout. b, Transcriptional fusion of P_{blc}-GFP in WT (6 biological replicates, 2,509 cells, mean = 0.050 AU, CV = 0.46) and $\Delta rpoS$ (4 bio. reps., 1,190 cells, mean = 0.025 AU, CV = 0.21). c, Similarly for P_{poxB}-GFP (WT: 5 bio. reps., 1,087 cells, mean = 0.12 AU, CV = 0.59; $\Delta rpoS$: 7 bio. reps., 1,463 cells, mean = 0.023 AU, CV = 0.17). Lines and shaded region are mean ± std dev, respectively.

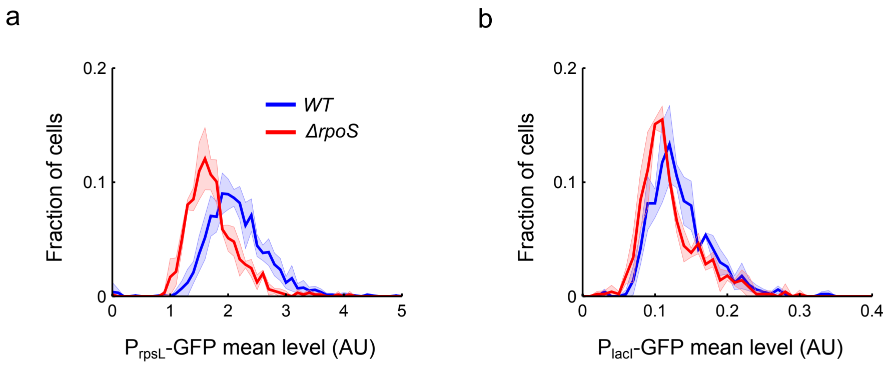

Supplementary Figure 3: Reporters of σ^{70} have distributions with lower coefficients of variation than RpoS reporters and distributions that are similar in *WT* and $\Delta rpoS$. a, Transcriptional fusion of P_{rpsL}-*GFP* in *WT* (5 bio. reps., 1,576 cells, mean = 2.1 AU, CV = 0.25) and $\Delta rpoS$ (3 bio. reps., 647 cells, mean = 1.7 AU, CV = 0.25). b, Similarly for P_{lacI}-*GFP* in *WT* (3 bio. reps., 503 cells, mean = 0.14 AU, CV = 0.31) and $\Delta rpoS$ (3 bio. reps., 497 cells, mean = 0.12 AU, CV = 0.34). Lines and shaded region are mean ± std dev, respectively.

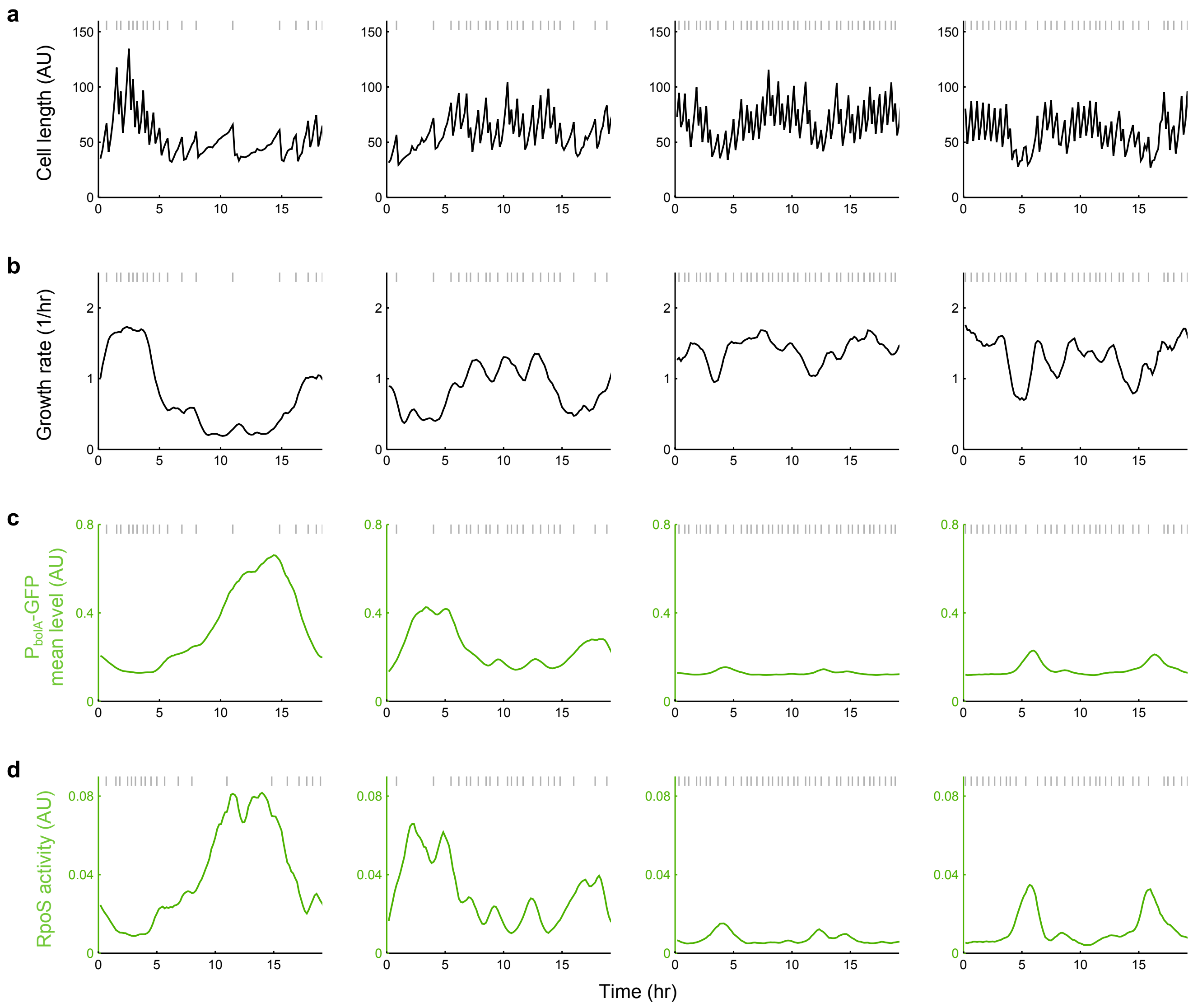

Supplementary Figure 4: Illustration of promoter activity computation for sample traces in Figure 2. a, Cell length (l). b, Growth rate is computed as the normalised numerical derivative of cell length: $g = \frac{1}{l}\frac{dl}{dt}$. c, Mean fluorescence per cell from the P_{bolA}-GFP reporter (M). d, Promoter (RpoS) activity is the rate of GFP production from the reporter due to RpoS, computed using $A = M(\frac{1}{l}\frac{dl}{dt} + p) + \frac{dM}{dt}$ (see Methods for derivation). Grey vertical lines indicate cell divisions.

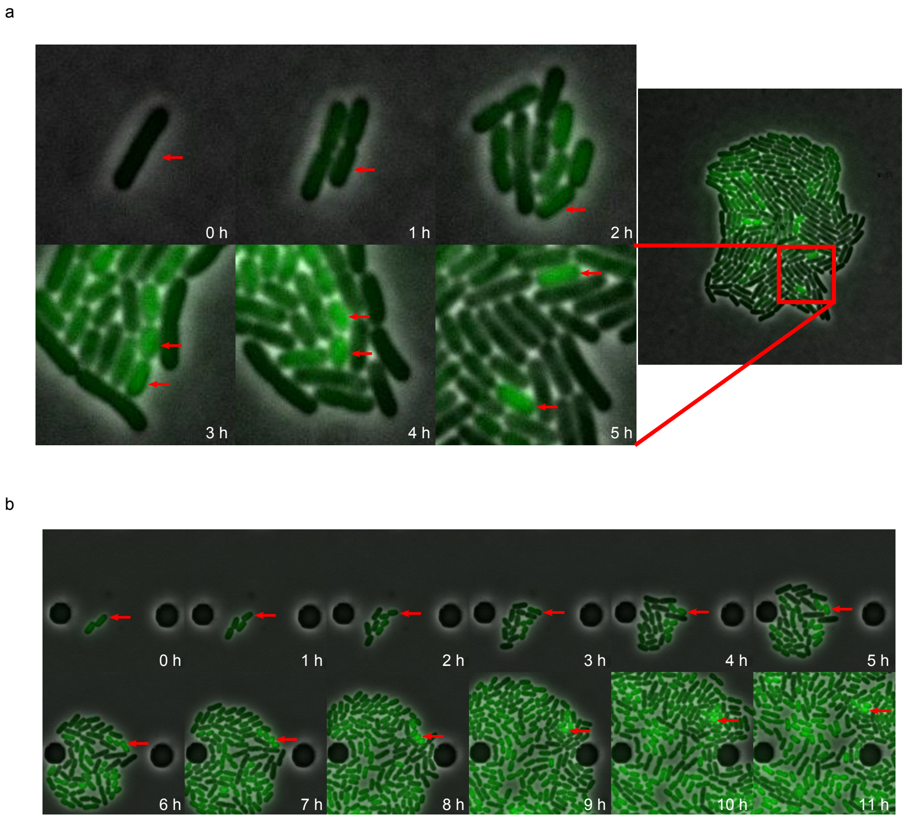

Supplementary Figure 5: RpoS pulsing is not an artefact of the Mother Machine microfluidic device environment. a, Sample movie montage of cells (MG1655 *WT* with P_{bolA}-*GFP*) grown on M9 agarose pads. A biological repeat showed similar effects. Red arrows indicate a lineage that grows slower than its neighbours and has high RpoS expression. A zoomed out image of the last frame illustrates the contrast in growth rates between the highlighted lineage and the remainder of the colony. b, Sample movie montage of cells (BW25113 *WT* with P_{bolA}-*GFP*) grown in the CellASIC microfluidic device. A biological repeat showed similar effects. The red arrows indicate a cell lineage that grows slower and has higher RpoS expression than its neighbours. In both (a) and (b) cells were imaged every 10 minutes and phase contrast and fluorescence channel ranges chosen for display.

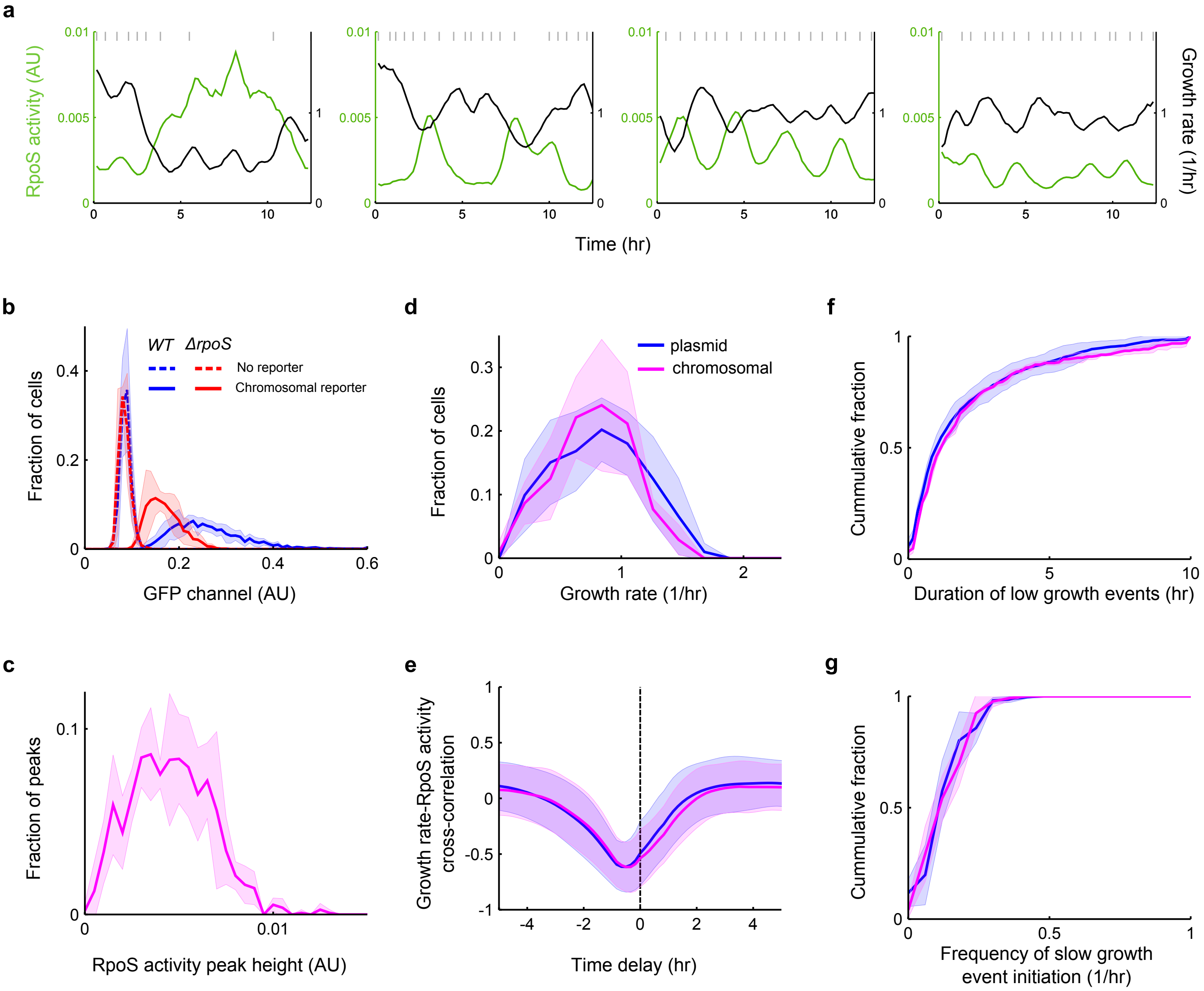

Supplementary Figure 6: Long-tailed RpoS distribution is not due to plasmid segregation effect, nor are the growth effects due to plasmid toxicity. a, Sample time traces of RpoS activity and growth rate for four mother cells with chromosomally integrated P_{bolA}-*GFP*. Grey vertical lines indicate cell divisions. b, Single cell measurements of bulk liquid culture measured with 3x higher exposure than for plasmid-borne reporter images: *WT* with chromosomally integrated P_{bolA}-*GFP*, 4 biological replicates, 2897 cells, mean = 0.26 AU, CV = 0.31; Δ*rpoS* with chromosomally integrated P_{bolA}-*GFP*, 4 biological replicates, 2039 cells, mean = 0.17 AU, CV = 0.21; *WT* with no reporter, 2 biological replicates, 1170 cells, mean = 0.087 AU, CV = 0.12; Δ*rpoS* with no reporter, 2 biological replicates, 954 cells, mean = 0.085 AU, CV = 0.15. c-g, *WT* with chromosomally integrated P_{bolA}-*GFP*; 4 technical replicates drawn from 2 bio. reps., 106 mother cells. The plasmid data in d-g is reproduced from elsewhere in this work for ease of comparison. Lines and shaded region are mean ± std dev, respectively. c, Pulse height distribution in Mother Machine experiments (990 peaks). d, Growth rate histogram at one frame (59) from all movies (104 cells). e, Cross-correlation between growth rate and RpoS activity. f, Duration distribution of low growth events (282 events). g, Distribution of frequency of entering low growth event (254 events).

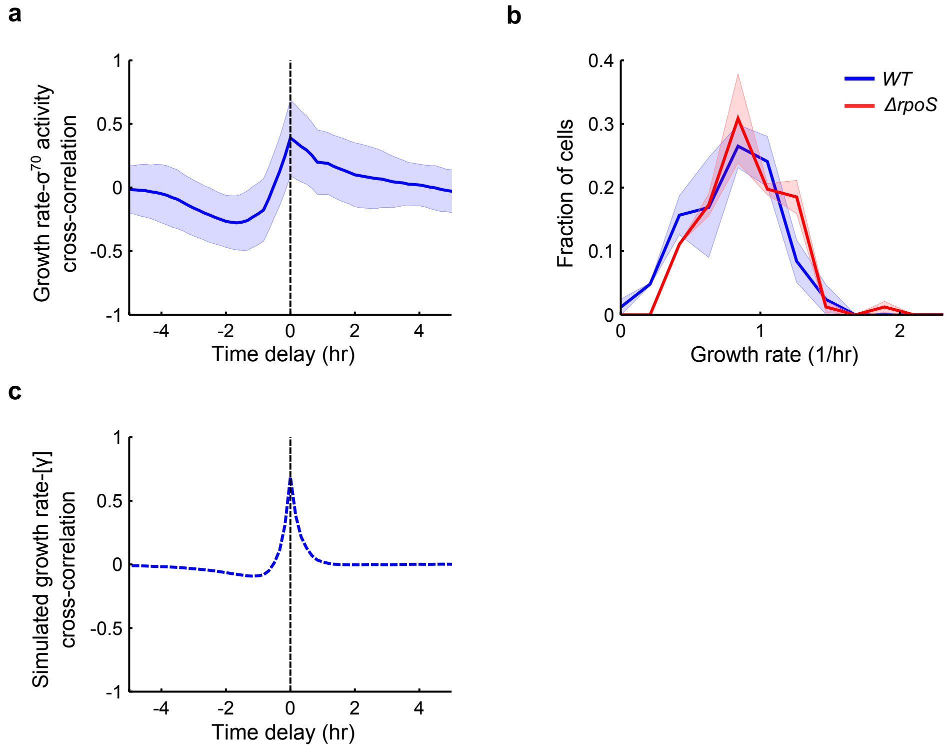

Supplementary Figure 7: Constitutive, σ^{70}, reporter is positively correlated with growth and high GFP expression does not affect growth rate distribution. Using P_{rpsL}*-GFP* in *WT* and $\Delta rpoS$. a, Cross-correlation between growth rate and σ^{70} activity in *WT* cells. b, Growth rate histogram for *WT* and $\Delta rpoS$ at one frame (59) from all movies. *WT*: 2 biological replicates, 83 mother cells; $\Delta rpoS$: 2 biological replicates, 81 mother cells. Lines and shaded region are mean $\pm$ std dev, respectively. c, Cross-correlation between simulated growth rate and γ concentration. Analysis from 1,000 simulations run for 500 hours; only the last 250 hours are used to avoid initial transients in the simulation.

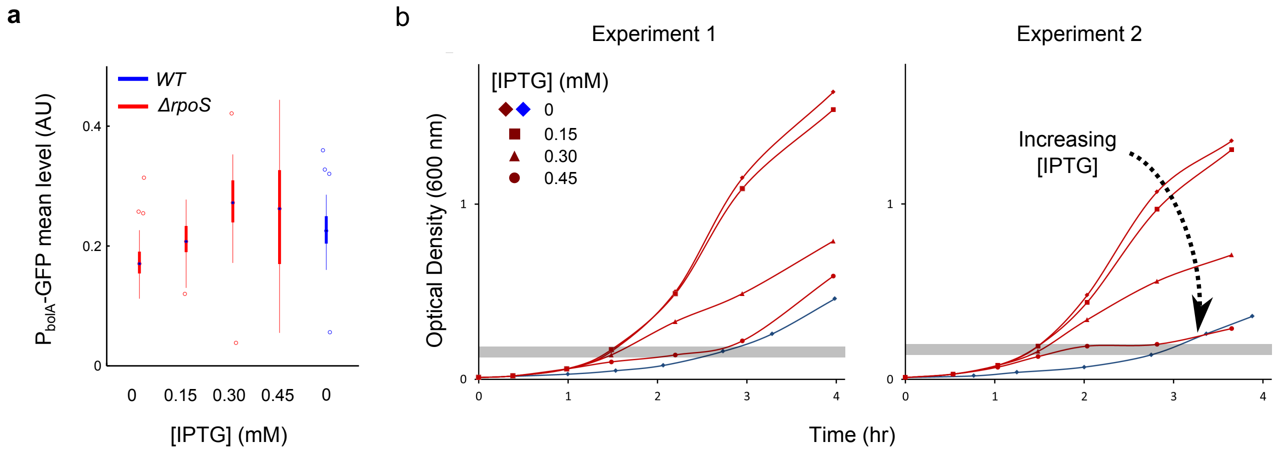

Supplementary Figure 8: Inducing RpoS over-expression reduces growth rate. a, Increased levels of IPTG induction produced increasing P_{bolA}-GFP levels (box plots, median ± 25th to 75th quartiles (box) ± full distribution (whiskers) ± outliers (points); $\Delta rpoS$+inducible construct: 0 mM IPTG, 136 cells, mean = 0.23 AU, CV = 0.17; 0.15 mM IPTG, 132 cells, mean = 0.21 AU, CV = 0.15; 0.30 mM IPTG, 137 cells, mean = 0.27 AU, CV = 0.19; 0.45 mM IPTG, 94 cells, mean = 0.25, CV = 0.39; WT+inducible construct: 0 mM IPTG, 115 cells, mean = 0.23 AU, CV = 0.17; data drawn from the shaded region of two experiments in (b). b, Bulk culture growth curves of $\Delta rpoS$+inducible construct under IPTG induction, and WT+inducible construct without induction.

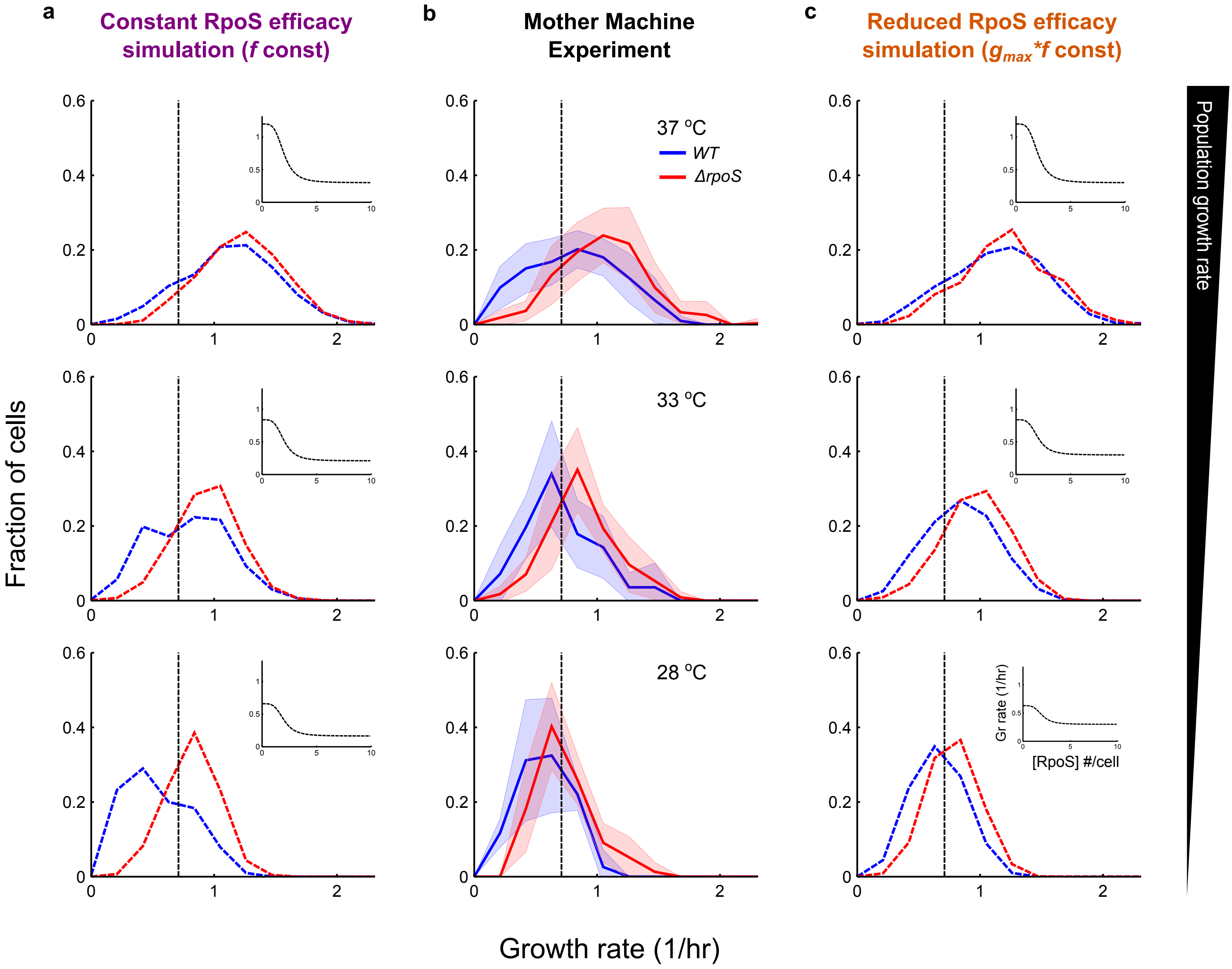

Supplementary Figure 9: The influence of RpoS on growth is attenuated as population growth rate decreases. a, Growth rate histograms for simulated *WT* and $\Delta rpoS$ at three population growth rates achieved by keeping f constant as g_{max} was reduced. Dashed black lines correspond to optimal survival threshold of 0.71/hr (Figure 4d). Insets: Hill functions of growth rate vs RpoS concentration. b, Experimental growth rate histograms for *WT* and $\Delta rpoS$ grown at three temperatures sampled at one frame (59) from all movies (mean ± std dev, 28°C and 37°C reproduced from main text; 33°C *WT*, 5 technical replicates drawn from 3 biological replicates, 56 mother cells; $\Delta rpoS$, 6 tech. reps. drawn from 3 bio. rep., 114 mother cells). c, Growth rate histograms for simulated *WT* and $\Delta rpoS$ with $f * g_{max}$ constant as g_{max} was reduced. Insets: same as (a). g_{max} values for the simulations were chosen such that *WT* population growth rates matched the experimentally observed population growth rates. For (a) and (c) 100 simulations were used for each condition, sampled every 24 hours, in the final 250 hours of 500 hour simulations.

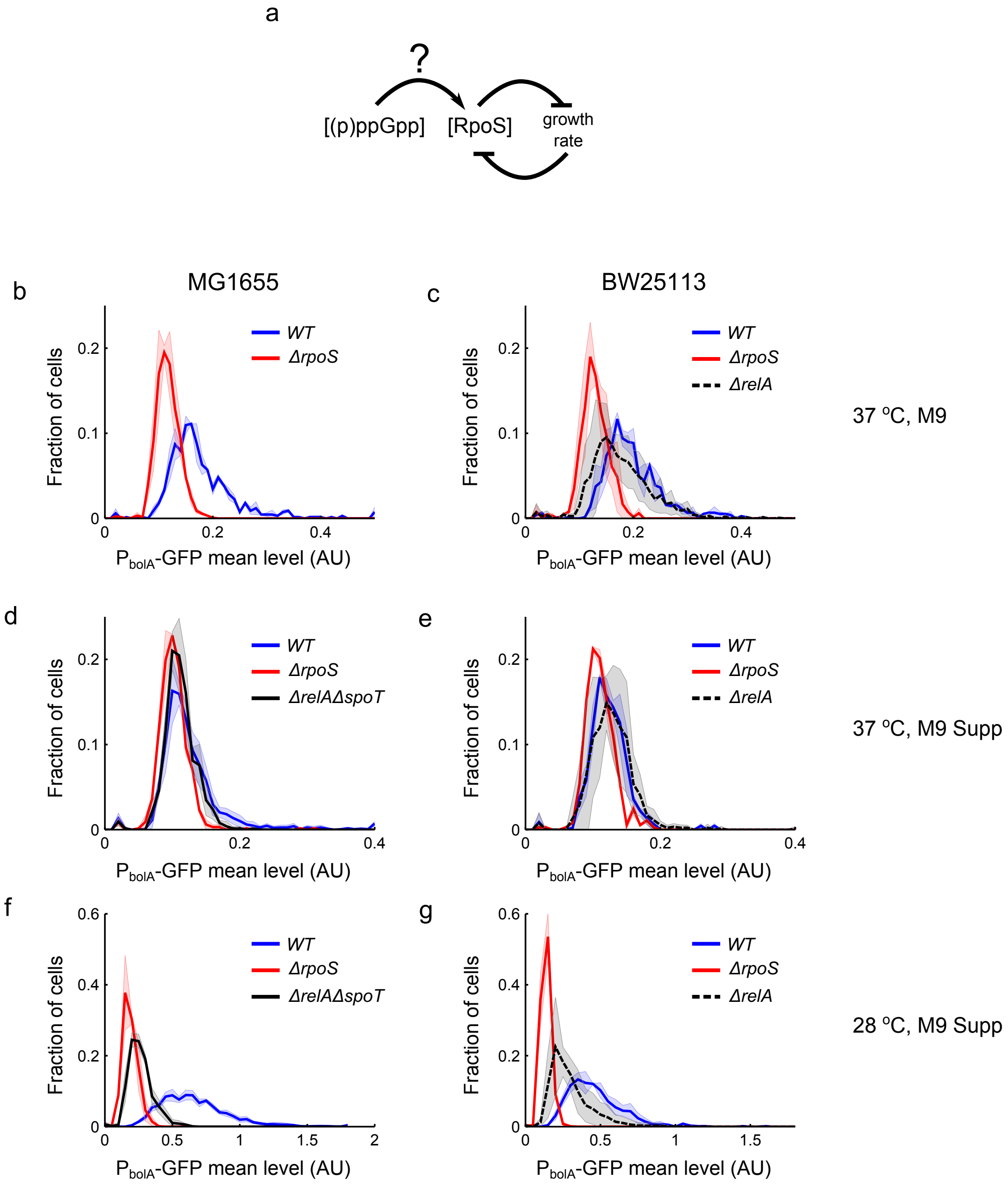

Supplementary Figure 10

(caption continued)

Supplementary Figure 10 caption, continued

(p)ppGpp does not abolish RpoS heterogeneity. a, Schematic illustrating the dynamic role (p)ppGpp could have on RpoS heterogeneity. b, c, Testing single gene knockout, Δ*relA*, the primary (p)ppGpp synthase, in standard conditions. b, *WT* strain used in this work, MG1655, and Δ*rpoS::kan* harbouring reporter with kanamycin resistance replaced with spectinomycin resistance (P_{bolA}-*GFP::spec*). *WT* (2 biological replicates, 666 cells, mean = 0.18 AU, CV = 0.37) and Δ*rpoS* (2 bio. reps., 1,246 cells, mean = 0.12 AU, CV = 0.18). c, The same in the *WT* strain of the Keio collection [2], BW25113. *WT* (2 bio. reps., 745 cells, mean = 0.20 AU, CV = 0.28), Δ*rpoS* (2 bio. reps., 658 cells, mean = 0.13 AU, CV = 0.21), and Δ*relA* (4 bio. reps., 1417 cells, mean = 0.18 AU, CV = 0.31). d and e, The same as (b) and (c) grown at 37°C in media supporting growth of the Δ*relA*Δ*spoT* double mutant in the MG1655 background [5]. d, MG1655 *WT* (6 bio. reps., 1974 cells, mean = 0.13 AU, CV = 0.46), Δ*rpoS* (2 bio. reps., 662 cells, mean = 0.10 AU, CV = 0.22), and Δ*relA*Δ*spoT* (4 bio. reps., 752 cells, mean = 0.11 AU, CV = 0.20) using the P_{bolA}-*GFP::kan* reporter in all three strains. e, BW25113 *WT* (2 bio. reps., 452 cells, mean = 0.12 AU, CV = 0.23), Δ*rpoS* (2 bio. reps., 372 cells, mean = 0.11 AU, CV = 0.19), and Δ*relA* (6 bio. reps., 894 cells, mean = 0.13 AU, CV = 0.25). f and g, The same as (d) and (e) but grown at 28°C to restore RpoS heterogeneity and so test the double mutant Δ*relA*Δ*spoT*. f, MG1655 *WT* (6 bio. reps., 4307 cells, mean = 0.66 AU, CV = 0.40), Δ*rpoS* (2 bio. reps., 1488 cells, mean = 0.19 AU, CV = 0.28), and Δ*relA*Δ*spoT* (4 bio. reps., 1070 cells, mean = 0.26 AU, CV = 0.34) using the P_{bolA}-*GFP::kan* reporter in all three strains. g, BW25113 *WT* (2 bio. reps., 713 cells, mean = 0.46 AU, CV = 0.40), Δ*rpoS* (2 bio. reps., 891 cells, mean = 0.14 AU, CV = 0.21), and Δ*relA* (6 bio. reps., 2738 cells, mean = 0.30 AU, CV = 0.46).

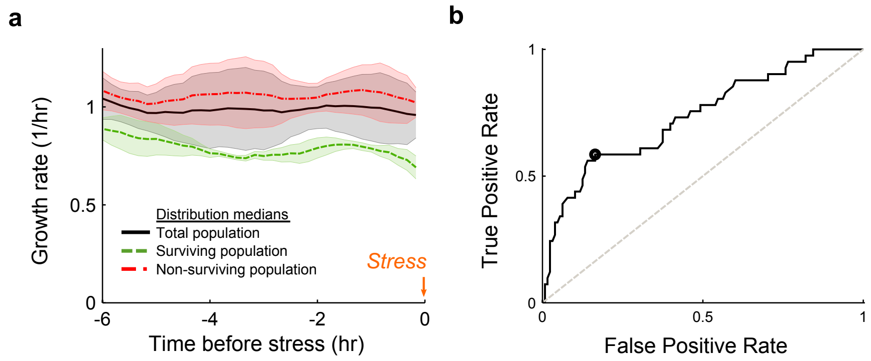

Supplementary Figure 11: Slow growing Δ*rpoS* cells survive oxidative stress. Cells were treated as in Figure 4. a, Median value of growth rate distributions for time points prior to stress application (t = 0), sorted according to survival (mean ± std dev, 5 technical replicates drawn from 3 biological replicates, 41 surviving cells, 128 non-surviving cells, 169 total mother cells). b, Receiver Operating Characteristic curve for growth rate (optimal threshold is 0.72/hr, Area Under Curve is 0.74).

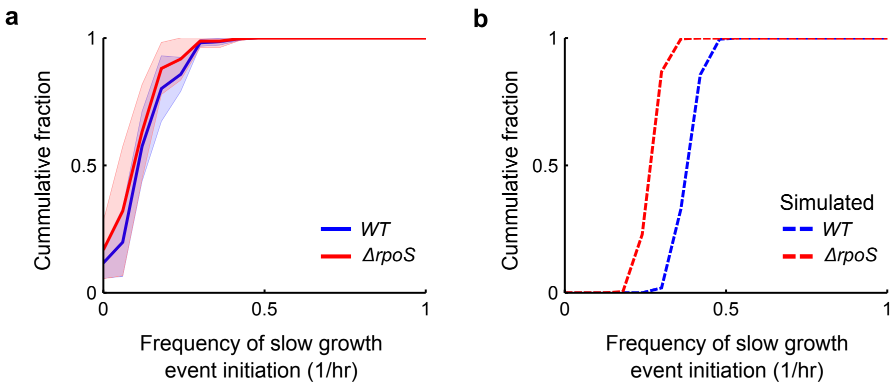

Supplementary Figure 12: Frequency of slow growth initiation is similar between *WT* and Δ*rpoS*. a, Experimental distributions of frequency of entering low growth event for *WT* and Δ*rpoS* (mean ± std dev; *WT*, 11 technical replicates drawn from 7 biological replicates, 507 mother cells, 862 events; Δ*rpoS*, 10 tech. reps. drawn from 6 bio. rep., 274 mother cells, 435 events). b, Same as (a) for simulations (1,000 simulations run for 500 hours, only the final 250 hours were used; *WT*, 102,149 events and Δ*rpoS*, 73,339 events).

Supplementary References

[1] A. Zaslaver, A. Bren, M. Ronen, S. Itzkovitz, I. Kikoin, S. Shavit, W. Liebermeister, M. G. Surette, and U. Alon, "A comprehensive library of fluorescent transcriptional reporters for Escherichia coli," *Nature Methods*, vol. 3, no. 8, pp. 623–628, 2006.

[2] T. Baba, T. Ara, M. Hasegawa, Y. Takai, Y. Okumura, M. Baba, K. A. Datsenko, M. Tomita, B. L. Wanner, and H. Mori, "Construction of Escherichia coli K-12 in-frame, single-gene knockout mutants: the Keio collection," *Molecular Systems Biology*, vol. 2, 2006.

[3] A. Harms, C. Fino, M. A. Sørensen, S. Semsey, and K. Gerdes, "Prophages and Growth Dynamics Confound Experimental Results with Antibiotic-Tolerant Persister Cells," *mBio*, vol. 8, no. 6, 2017.

[4] G. Chen and H. E. Schellhorn, "Controlled induction of the RpoS regulon in Escherichia coli, using an RpoS-expressing plasmid," *Canadian Journal of Microbiology*, vol. 49, no. 12, pp. 733–740, 2003.

[5] K. Potrykus, H. Murphy, N. Philippe, and M. Cashel, "ppGpp is the major source of growth rate control in E. coli," *Environmental Microbiology*, vol. 13, no. 3, pp. 563–575, 2010.
