# Supplementary material for: Escherichia coli can survive stress by noisy growth modulation
Source: Nat Commun. 2018 Dec 17;9:5333. doi: 10.1038/s41467-018-07702-z (PMC6297224; doi:10.1038/s41467-018-07702-z)
Supplement: Supplementary file 2 — Description of Additional Supplementary Files [file 41467_2018_7702_MOESM2_ESM.pdf]

Description of Additional Supplementary Files

File Name: Supplementary Movie 1
Description: Sample movie of P_{bolA}-*GFP* plasmid reporter in *WT* cells growing in the Mother Machine. Pulses of P_{bolA}-*GFP* coupled to slow growth rate can be observed. Phase contrast (grey) and fluorescence channel (green) ranges chosen for display. The time between frames in this movie is 10 minutes.

File Name: Supplementary Movie 2
Description: Sample movie of micro-colony grown from a single cell illustrating RpoS and growth pulsing. P_{bolA}-*GFP* plasmid reporter in *WT* growing on agarose pads containing M9 media. Phase contrast (grey) and fluorescence channel (green) ranges chosen for display. The time between frames in this movie is 10 minutes.

File Name: Supplementary Movie 3
Description: Sample movie of cells in an alternative microfluidic device, the CellASIC, illustrating RpoS and growth pulsing. P_{bolA}-*GFP* plasmid reporter in *WT* cells (BW25113). Phase contrast (grey) and fluorescence channel (green) ranges chosen for display. The time between frames in this movie is 10 minutes.

File Name: Supplementary Movie 4
Description: Sample movie of P_{blc}-*GFP* plasmid reporter in *WT* cells growing in the Mother Machine. Pulses of P_{blc}-*GFP* coupled to slow growth rate can be observed. Phase contrast (grey) and fluorescence channel (green) ranges chosen for display. The time between frames in this movie is 10 minutes.

File Name: Supplementary Movie 5
Description: Sample movie of P_{poxB}-*GFP* plasmid reporter in *WT* cells growing in the Mother Machine. Pulses of P_{poxB}-*GFP* coupled to slow growth rate can be observed. Phase contrast (grey) and fluorescence channel (green) ranges chosen for display. The time between frames in this movie is 10 minutes.
